# Predicting and understanding law-making with word vectors and an ensemble model

**DOI:** 10.1371/journal.pone.0176999

**Published:** 2017-05-10

**Authors:** John J. Nay

**Affiliations:** 1 School of Engineering, Vanderbilt University, Nashville, TN, United States of America; 2 Program on Law and Innovation, Vanderbilt Law School, Nashville, TN, United States of America; Tianjin University, CHINA

## Abstract

Out of nearly 70,000 bills introduced in the U.S. Congress from 2001 to 2015, only 2,513 were enacted. We developed a machine learning approach to forecasting the probability that any bill will become law. Starting in 2001 with the 107th Congress, we trained models on data from *previous* Congresses, predicted all bills in the *current* Congress, and repeated until the 113th Congress served as the test. For prediction we scored each sentence of a bill with a language model that embeds legislative vocabulary into a high-dimensional, semantic-laden vector space. This language representation enables our investigation into which words increase the probability of enactment for any topic. To test the relative importance of text and context, we compared the text model to a context-only model that uses variables such as whether the bill’s sponsor is in the majority party. To test the effect of changes to bills after their introduction on our ability to predict their final outcome, we compared using the bill text and meta-data available at the time of introduction with using the most recent data. At the time of introduction context-only predictions outperform text-only, and with the newest data text-only outperforms context-only. Combining text and context always performs best. We conducted a global sensitivity analysis on the combined model to determine important variables predicting enactment.

## 1 Introduction

The U.S. legislative branch creates laws that impact the lives of hundreds of millions of citizens. For example, the Patient Protection and Affordable Care Act (ACA) significantly affected the health care industry and individuals’ health insurance coverage. Bills often consist of hundreds of pages of dense legal language. In fact, the ACA is more than 900 pages long. There are thousands of bills under consideration at any given time and only about 4% will become law. Furthermore, the number of bills introduced is trending upward (see S1 Appendix), exacerbating the problem of determining what text is relevant. Given the complexity, length, and vast quantity of bills, a machine learning approach that leverages bill text is well-suited to forecast bill success and identify the important predictive variables. Despite rapid advancement of machine learning methods, it’s difficult to outperform naive forecasts of rare events because of inherent variability in complex social processes [1] and because relationships learned from historical data can change without warning and invalidate models applied to future circumstances.

Due to the complexity of law-making and the aleatory uncertainty in the underlying social systems, we predict enactment probabilistically. It’s important to make *probabilistic* predictions for high consequence events because even small changes in probabilities for events with extreme implications can have large expected values. For instance, the 2009 stimulus bill cost $831 billion so even a 0.1 change in the predicted probability of this bill corresponds to a $83.1 billion dollar change in the expected value (the probability of an event multiplied by its consequences). Probabilities provide much more information than a simple “enact” or “not enact” prediction. Model performance metrics that don’t use probabilities, such as accuracy, are not suitable measures of rare event predictive ability. For instance, a blunt “never enact” model has a seemingly impressive 96% accuracy rate on this data but incorrectly classifies all the enacted bills with incalculable effects on society.

Forecasting model performance should be estimated using multiple metrics on large amounts of test data measured *after* the data that was used to train the model. We trained models on Congresses prior to the Congress predicted, which simulated real-time deployment across 14 years and 68,863 bills. Starting with the 107th Congress (2001–2003), models were sequentially trained on data from *previous* Congresses and tested on all bills in the *current* Congress. This was repeated seven times until the most recently completed Congress—the 113th (2013–2015)—served as the test. To estimate performance, we compared a baseline model to our models across three performance measures that leverage predicted probabilities.

Although previous research found that bill text was useful for predicting whether bills will survive committee [2] and for predicting roll call votes [3, 4], these authors tested their models on much less data than we do and predicted more frequently observed events: getting out of committee is more common than being enacted and bills up for vote are a small subset of all bills introduced. It’s not clear whether utilizing text models trained on previous Congresses will improve predictions of enactment of bills introduced in future Congresses beyond the predictive power of sponsorship, committee and other non-textual data. Text is noisy and completely different topics can be found within the same bill [5]. However, we hypothesized that there are unique semantic and syntactic signatures that consistently differentiate successful bills. Our second hypothesis was concerned with the changes to bills over their lives. Some bills, e.g. the ACA, are only a few pages when introduced but are hundreds of pages when enacted. However, 87% of bill texts don’t change after being introduced because they don’t progress further in the law-making process. We hypothesized that using the most recently available version of bill text and metadata would lead to stronger predictive performance for text and context models. To test these hypotheses, we designed an experiment across two primary dimensions: *data type* (text-only, text and context, or context-only) and *time* (using oldest or newest bill data).

Analyzing a model that makes successful ex ante predictions can be more informative than ex-post interpretations of socio-political events (outside experiment-like settings) due to the over-fitting that plagues most modeling of observational data [6]. However, because highly predictive models are often designed with only predictive power in mind, they rarely provide clear insights into relationships between predictor variables and the predicted outcome. When estimates of these relationships are provided for non-linear models, they are almost always measures of only magnitudes of the effects of predictor variables and not also the directions of the effects. Our work is not limited to raw predictive power. We estimate the *direction and magnitude* of the effect of each predictor variable in the model on the predicted probability of enactment. Furthermore, the text model reveals which words are more associated with enactment success.

## 2 Methods and data

### 2.1 Model training

#### 2.1.1 Word vectors and inversion of language models

**Fig 1:**
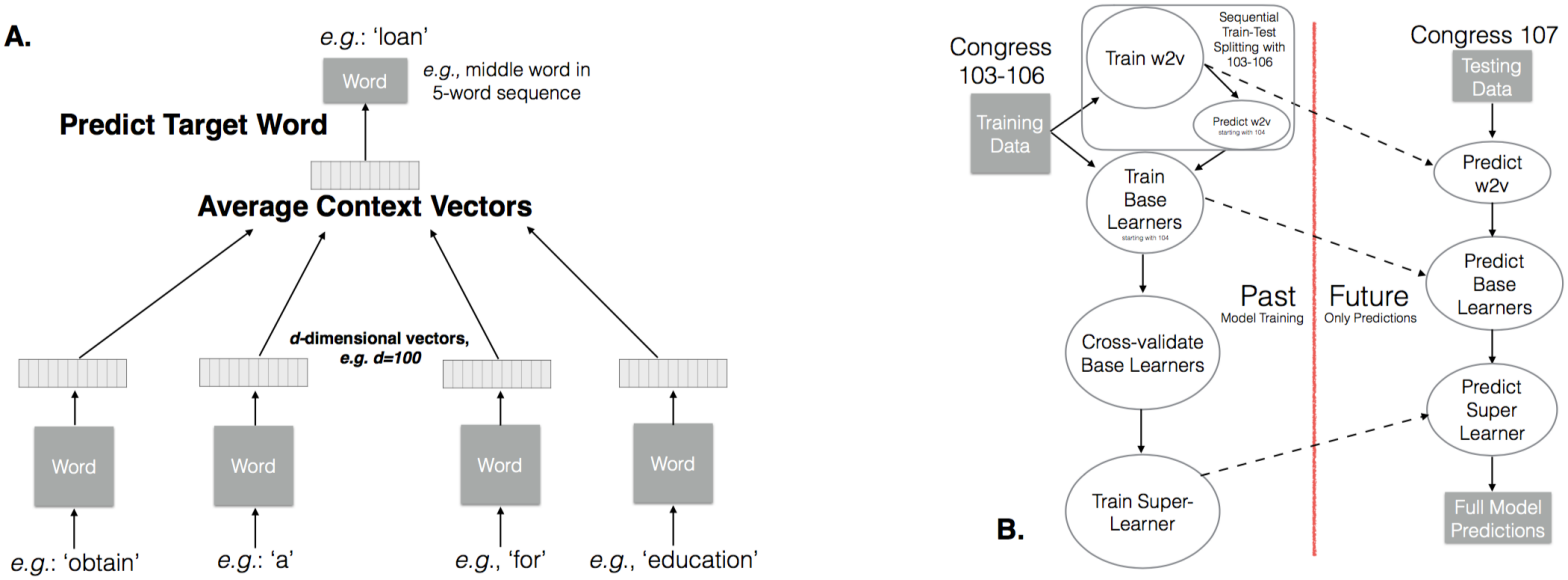
**A.** The neural network-based training algorithm used to obtain word vectors [10]. Parameters are updated with stochastic gradient descent and we use a binary Huffman tree to implement efficient softmax prediction of words. See S1 Appendix for description of hyper-parameters. **B.** Model training and testing. This process is completed and then we advance one Congress.

Continuous-space vector representations of words can capture subtle semantics across the dimensions of the vector [7]. To learn these representations, a neural network model predicts a target word with the mean of the representations of the surrounding words (e.g. vectors for the two words on either side of the target word in Fig 1A). The prediction errors are then back-propagated through the network to update the representations in the direction of higher probability of observing the target word [8, 9]. After randomly initializing representations and iterating this process over many word pairings, words with similar meanings are eventually located in similar locations in vector space as a by-product of the prediction task, which is called word2vec [8].

The only pre-processing we applied to text was removal of HTML, carriage returns, and whitespace, and conversion to lower-case. Then inversion of distributed language models was used for classification as described in [11]. Distributed language models—mappings from words to **R**^*d*^ obtained by leveraging word co-occurrences—were separately fit to the sub-corpora of successful and failed bills by applying word2vec. Each sentence of a testing bill was scored with each trained language model and Bayes’ rule was applied to these scores and prior probabilities for bill enactment to obtain posterior probabilities. The proportions of bills enacted in the same chamber as the predicted bill in all *previous* Congresses were used as the priors. The probabilities of enactment were then averaged across all sentences in a bill to assign an overall probability.

#### 2.1.2 Tree-based models

Trees are decision rules that divide predictor variable space into regions by choosing variables and their threshold values on which to make binary splits [12]. A tree model can learn interactions between predictors, unlike linear models where interactions must be manually specified, and is generally robust to the inclusion of variables unrelated to the outcome. A *gradient boosted machine* (GBM) improves an ensemble of weaker base models, often trees, by sequentially adjusting the training data based on the residuals of the preceding models [13]. A *random forest* randomly samples observations from training data and grows a tree on each sample, forcing each tree to consider randomly selected sets of predictor variables at each split to reduce correlation between trees [14]. GBMs and random forests can both learn non-linear functions but have different strengths: in general, random forests are more robust to outliers while GBMs can more effectively learn complex functions. A regularized logistic regression (elastic-net) with hyper-parameters (*α* = 0.5 and *λ* = 1e-05) is also estimated to gain a complementary linear perspective [15].

Using the predictions from the inversion of the word vector language model (as described in Section 2.1.1.) as features allows the training process to learn interactions between contextual variables and textual probabilities. Additionally, the sensitivity analysis can then estimate the impact of text predictions on enactment probabilities along with the contextual predictors, controlling for the effect of the probability of the bill text when estimating non-textual effects.

#### 2.1.3 Ensemble stacking

Random forests and GBMs combine weak learners to create a strong learner. Stacking combines strong learners to create a stronger learner. A cross-validation stacking process on the training data is used to learn a combination of the three base models to form a meta-predictor [16, 17]. Out-of-fold cross-validation predictions are made on the training data with the three base learners described above in Section 2.1.2 (the gradient boosted machine model, the random forest model, and the regularized logistic regression model). These predictions and the outcome vector are used to train the meta-learner, a regularized logistic regression with non-negative weights. Weights are forced to be non-negative because we assume all predictors should positively contribute. This entire learning process is conducted on data from prior Congresses. The model is applied to test data by making predictions with base learners and feeding those into the meta-learner (Fig 1B).

### 2.2 Model performance

We use the two most frequently applied binary classification probability scoring functions: the log score and the brier score (see S1 Appendix). For both, if a model assigns high probability to a failed bill it’s penalized more than if it was less confident and if a model assigns high probability to an enacted bill it is rewarded more than if it wasn’t confident. A receiver operating characteristic curve (ROC) is built from points that correspond to the true positive rate at varying false positive rate thresholds with the model’s predictions sorted by the probability of the positive class (enacted bill) [18]. Starting at the origin of the space of true positive rate against false positive rate, the prediction’s impact on the rates results in a curve tracing vertically for a correct prediction and horizontally for an incorrect prediction. A perfect area under the ROC curve (AUC) is 1.0 and the worst is 0.5. AUC rewards models for being discriminative throughout the range of probabilities and is more appropriate than accuracy for imbalanced datasets.

### 2.3 Analysis

#### 2.3.1 Text model similarity analysis

We train language models with word2vec for enacted House bills, failed House bills, enacted Senate bills, and failed Senate bills and then investigate the most similar words within each of these four models to word vector combinations representing topics of interest. That is, for each of the four models, return a list of most similar words: $\arg\max_{v*\in V_{1:N}}cos(v*,\frac{1}{W}\;\sum_{i=1}^{W}\;w_{i}\times s_{i})$, where *w*_*i*_ is one of *W* word vectors of interest, *V*_1:*N*_ are the *N* most frequent words in the vocabulary of *M* words (rare words are retained to train the model, but *N* is set to less than *M* to exclude rare words during model analysis) excluding words corresponding to the *W* query vectors, *s*_*i*_ is *1* or *-1* for whether we are positively or negatively weighting *w*_*i*_, and $cos\left(a,b\right)=\frac{\sum_{i=1}^{d}\;a_{i}\times b_{i}}{\sqrt{\sum_{i=1}^{d}\;a_{i}^{2}}\sqrt{\sum_{i=1}^{d}\;b_{i}^{2}}}$. For ease of comparison across enacted and failed categories we also remove words the two have in common.

#### 2.3.2 Full model sensitivity analysis

We conduct a sensitivity analysis on our model of the legislative system by varying inputs to the model and measuring the effect on the output. If input values are varied one at a time, while keeping the others at “default values,” sensitivities are conditional on the chosen default values [19]. There are no sensible default values for the predictor variables. Instead of using default values of variables, we use empirically observed predictor variables and we predict the enactment of all the bills from Congresses 104–112. These predictions are a vector of predicted probabilities. The empirical predictors variable data and these associated predicted probabilities create a sufficiently large yet realistic set of observations for a global sensitivity analysis to determine the modeled effects of the predictors variables on the probability of enactment.

Next, we expand the factor variables out so each level is represented in the design matrix as a binary indicator variable. This allows us to estimate the effect of each level of a factor, e.g. the 39 subject categories. We add interaction terms between the Chamber and bill characteristics, e.g. whether the bill originated in the Senate and the number of characters, to estimate these interaction effects potentially automatically learned by the tree models. Finally, we estimate the relationship between the resulting matrix of input values and the vector of predicted probability outputs with a partial rank correlation coefficient (PRCC) analysis, which estimates the correlation between an input variable and the predicted probability of bill enactment, discounting the effects of the other inputs and allowing for potentially non-linear relationships by rank-transforming the data before model estimation [20, 21]. Partial correlation controls for the other predictor variables, *Z*, by computing the correlation between the *residuals* of regressing the predictor of interest, *x*, on *Z* and the *residuals* of regressing the outcome (predicted probability of enactment) on *Z*. The PRCC analysis is bootstrapped 1,000 times to obtain 95% confidence intervals.

### 2.4 Data

We include all House and Senate bills and exclude simple, joint, and concurrent resolutions because simple and concurrent resolutions do not have the force of law and joint resolutions are very rare. We downloaded all bill data (from the 103rd Congress through the 113th Congress) other than committee membership from govtrack.us/developers/data, which is created by scraping THOMAS.gov. We downloaded committee membership data from web.mit.edu/17.251/www/data_page.html [22, 23].

There is often more than one version of the full text for each bill. In order to create a forecasting problem that predicts enactment as soon as possible, the earliest dated full text is used, which is, for more than 99% of the bills in the testing data, the text as it was introduced. To understand how much predictive power newer versions add, we collect the most recent version of each bill, which is, for 87% of the bills in the testing data, the version as introduced. Bills can change dramatically between the time of their introduction and the time of the last action taken on them. H.R. 3590 in the 111th Congress, was a short bill on housing tax changes for service members when it was introduced, and shortly before it was enacted it was the 906-page Affordable Care Act. H.R. 34 in the 114th Congress was originally introduced as the Tsunami Warning, Education, and Research Act and was about 30 pages long. Shortly before it was enacted, H.R. 34 was the 312-page 21st Century Cures Act.

The full text of all introduced bills is only available starting with the 103rd Congress (1993–1995) and therefore this is the first Congress used to train language models. The 104th Congress is the first used to train the base models of the ensemble because they require the language model predictions and the language models need the 103rd for training. The 107th Congress (2001–2003) is the first to serve as a testing Congress because the full model needs multiple Congresses worth of data for training. We used the list of predictor variables from [2] as a starting point for designing our feature set.

The following variables capture characteristics of a bill’s sponsor and committee(s):

- *region*: region corresponding to state the sponsor represents (5 levels).
- *sponsorPartyProp*: proportion of chamber in sponsor’s party (min: 0, median: 0.51, max: 0.59).
- *sponsorTerms*: number of terms sponsor has served in Congress (only up to Congress being predicted to ensure model is only using data that would have been available at that time, min: 1, median: 6, max: 30).
- *committeeSeniority*: mean length of time sponsor has been on the committees the bill is assigned to (min: 0, median: 0, max: 51). If not on committee, assigned 0.
- *committeePosition*: out of any leadership position of sponsor on any committee bill is assigned to, lowest number on the “leadership codes” list in S1 Appendix (11 levels, e.g. Chairman).
- *NotMajOnCom*: binary for whether sponsor is (*i*) *not* in majority party and (*ii*) on first listed committee bill is assigned to.
- *MajOnCom*: binary for whether sponsor is (*i*) in majority party and (*ii*) on first listed committee bill is assigned to.
- *numCosponsors*: number of co-sponsors (for oldest—min: 0, median: 2, max: 378; for newest—min: 1, median: 6, max: 432).

The following variables capture political and temporal context of bills:

- *session*: Session (first or second) of Congress that corresponds to full text date, almost always the date bill was introduced for oldest data (for oldest—proportion in first session: 0.64; for newest—proportion in first session: 0.6).
- *house*: binary for whether it’s a House bill.
- *month*: month bill is introduced.

The following variables capture aspects of bill content and characteristics:

- *subjectsTopTerm*: official top subject term (36 levels).
- *textLength*: number of characters in full text (for oldest—min: 119, median: 5,340, max: 2,668,424; for newest—min: 113, median: 5,454, max: 3,375,468).

## 3 Results

### 3.1 Prediction experiments

Five models are compared across the two time conditions. *w2v* is the scoring of full bill text with an inversion of word2vec-learned language representations [11]. We take this approach to textual prediction because it provides the capacity to conduct a semantic similarity text analysis across enacted and failed categories and can predict which sentences of a bill contribute most to enactment. *w2vTitle* is title-only scoring with the same method. *GLM* is a regularized non-negative generalized linear model (GLM) meta-learner over an ensemble of a regularized GLM, a gradient boosted machine and a random forest, which each use only the contextual variables (see Data section). *w2vGLM* is the same as *GLM* except the *w2v* and *w2vTitle* predictions are added as two more predictor variables for the three base learners. These are compared to a baseline, *null*, that uses the proportion of bills enacted in the same chamber as the predicted bill across all previous Congresses as the predicted probability. For instance, the proportion of bills enacted in the Senate from the 103rd to the 110th Congress was 0.04 and so this is the *null* predicted probability of enactment of a Senate bill in the 111th Congress. It’s important to use Chamber-specific rates to improve *null* performance because bills originating in the House have a higher enactment rate.

**Fig 2:**
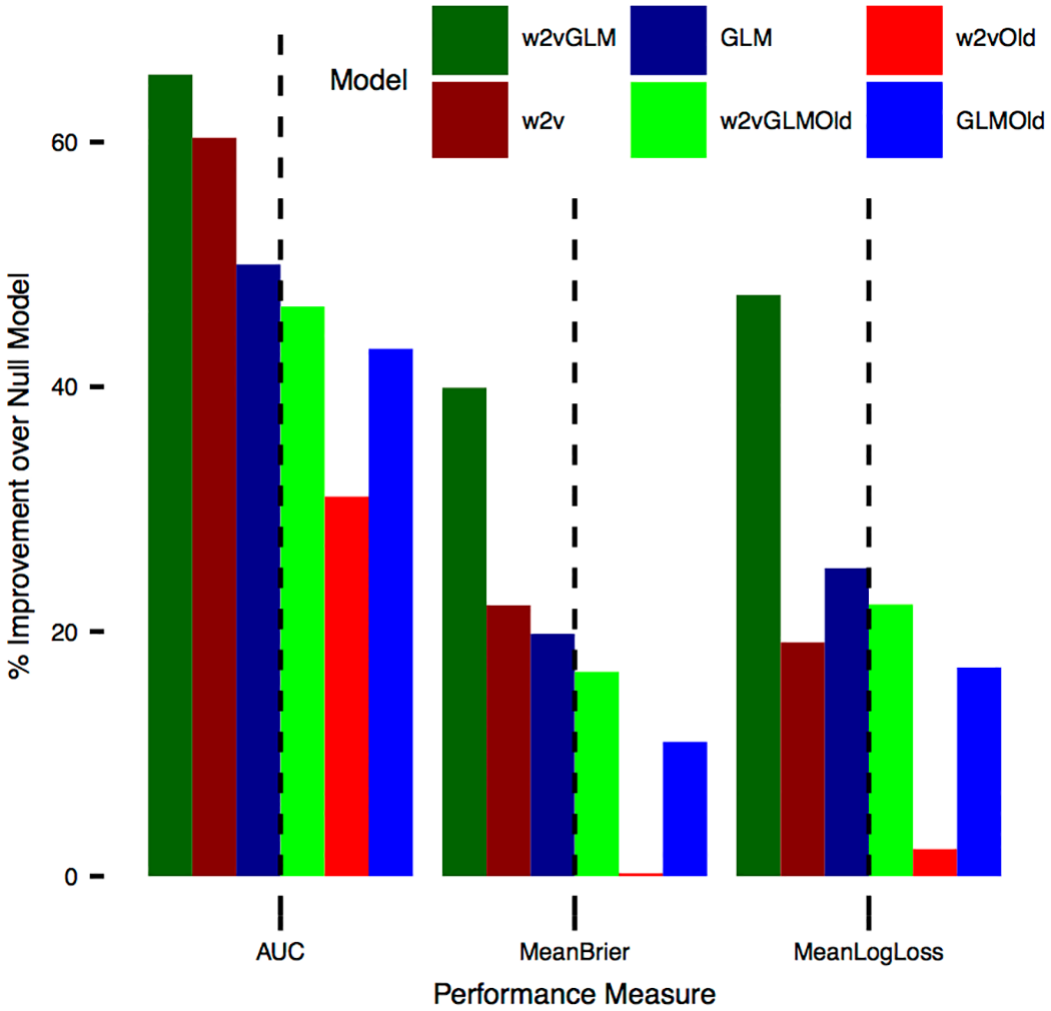
Percent improvement of each model over *null* (n = 68,863). Dashed lines separate newest and oldest data within each measure. Because *w2vTitle* has infinite log loss it’s not included in the figure.

Using only text outperforms using only context on two of three performance measures (AUC and Brier) for the newest data, while using only context outperforms only text on three measures for the oldest data (Fig 2). Using text and context together, *w2vGLM*, outperforms all competitors on all measures for newest and oldest data (Table 1). When predicting enactment with the newest bill text and the updated number of cosponsors, text length and session, both models improved but *w2vGLM* and *w2v* improved dramatically. *w2vGLM* has the highest AUC, *w2v* has the second highest for predictions with new data and *GLM* has the second highest for predictions with old data.

**Table 1:** Model performance comparison (n = 68,863). Lower mean brier score (MeanBrier) and mean log loss (MeanLogLoss) is better and higher AUC is better. *w2vTitle* has infinite log loss due to making predictions with 0 and 1 probabilities. Two-sample t-tests with alternative hypotheses that *w2vGLM* outperforms its closest competitors are significant with p-values of 3.02e-53 (log loss newest data), 3.65e-26 (brier loss newest data), 9.73e-04 (log loss oldest data) and 5.36e-03 (brier loss oldest data).

Model	AUC	MeanBrier	MeanLogLoss
w2vGLM	0.96	0.021	0.083
w2v	0.93	0.027	0.127
GLM	0.87	0.028	0.118
w2vTitle	0.81	0.049	Inf
Null	0.58	0.035	0.157
w2vGLMOld	0.85	0.029	0.122
w2vOld	0.76	0.035	0.154
GLMOld	0.83	0.031	0.131
w2vTitleOld	0.8	0.047	Inf

**Fig 3:**
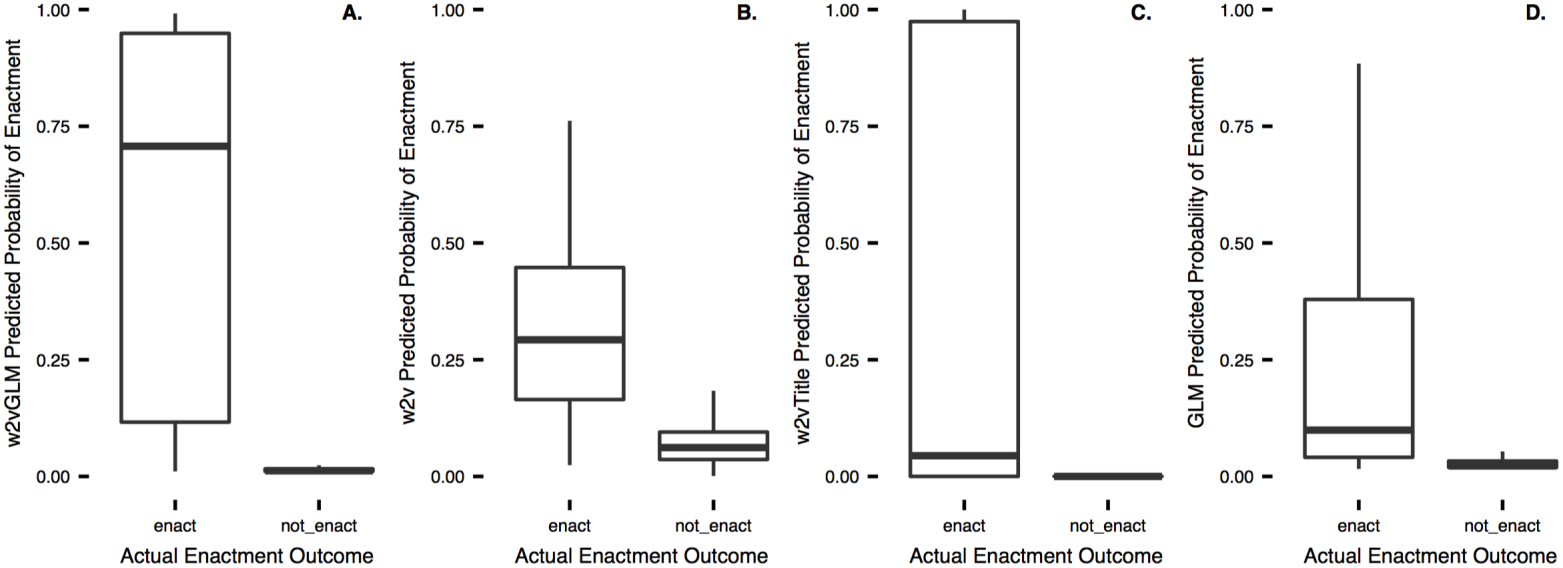
Boxplots (n = 68,863) of predicted probabilities of enacted and failed bills for *w2vGLM* (A.), *w2v* (B.), *w2vTitle* (C.), *GLM* (D.). The boxes are the inter-quartile ranges (IQRs) of the predicted probabilities, the bold line is the median, the whiskers extend from ends of IQR to +/− 1.5 * *IQR*.

Predicted probabilities of *w2vGLM* range from 0.01 to 0.99. In fact, the majority of the predicted probabilities are near 0 and 1 (S1 Appendix). This is impressive given that it still maintains overall high performance on log and brier scoring, which significantly penalize models for high probability predictions on the wrong side of 0.5. The central tendencies of the predicted probabilities (mean = 0.05, and median = 0.01) are close to the observed frequency of bill enactment, 0.04. The median of the predicted probabilities where the true outcome was not enact (0.01) is much lower than the median of the predicted probabilities where the true outcome was enact (0.71) (Fig 3A). The *w2v* predicted probabilities (Fig 3B) demonstrate that with just the text of the bills, the model can make probabilistic predictions that discriminate between enacted and failed bills, providing credibility to our textual semantic similarity analysis. In contrast, the title-only (Fig 3C) and context-only (Fig 3D) models poorly discriminate.

We conduct an error analysis (see S1 Appendix) and find that, across all bill subjects, *w2vGLM* and *w2v* both have the highest log loss on two categories of low economic importance: Commemorations; and Arts, Culture, Religion. As a final investigation of model performance, we explore the *w2vGLM* predictions for the two most significant bills in the past century: the ACA and the American Recovery and Reinvestment Act of 2009 (ARRA). The density of the predicted probabilities for all bills is peaked around 0.01 (S1 Appendix) and the predicted probabilities for the ACA and ARRA were > 0.5 (Table 2). None of the 296 other bills with “Patient Protection and Affordable Care Act” in the title were enacted. These bills (all official titles listed in S1 Appendix) attempted to amend or repeal the ACA, which could have had significant economic effects. In 2012, the Congressional Budget Office estimated that H.R. 6079, the Repeal of Obamacare Act, would cause a $109 billion net increase in federal deficits [24]. *w2vGLM*’s predicted probabilities for these failed attempts are much more useful than *null*’s (Table 2). 96% of the (unsuccessful) bills to repeal and amend the ACA have *lower* predicted probabilities of enactment from *w2vGLM* than from *null*.

**Table 2:** Predicted probabilities of enactment for key bills. Probabilities increased between old and new forecasts for the two enacted bills, and the mean of the probabilities for the failed bills decreased.

ShortTitle	ForecastNew	ForecastOld	BaselineForecast
ACA	0.6	0.23	0.05
Failed Amend Repeal	0.02	0.03	0.05
ARRA	0.55	0.52	0.05

### 3.2 Analysis

Now that we have a model validated on thousands of predictions, we analyze it to better understand law-making. With our language models, we create “synthetic summaries” of hypothetical bills by providing a set of words that capture any topic of interest. Comparing these synthetic summaries across chamber and across Enacted and Failed categories uncovers textual patterns of how bill content is associated with enactment. The title summaries are derived from investigating similarities within *w2vTitle* and the body summaries are derived from similarities within *w2v*. Distributed representations of the words in the bills capture their meaning in a way that allows semantically similar words to be discovered. Although bills may not have been devoted to the topic of interest within any of the four training data sub-corpora, these synthetic summaries can still yield useful results because the queried words have been embedded within the semantically structured vector space along with all vocabulary in the training bills. This is important for a topic, such as climate change, with little or no relevant enacted legislation.

**Fig 4:**
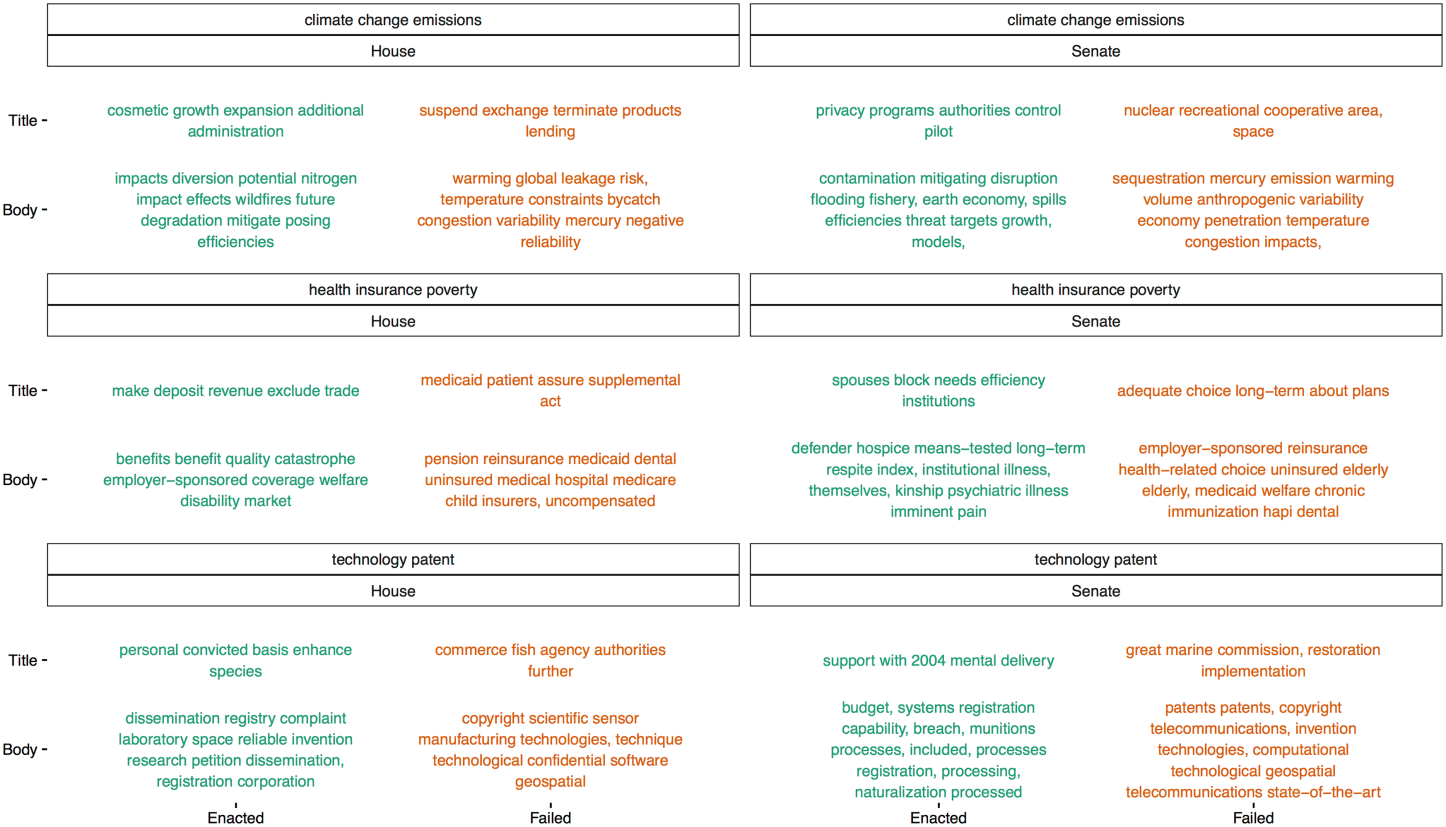
Synthetic summary bills for three topics across enacted and failed and house and Senate categories.

To demonstrate the power of our approach, we investigated the words that best summarize “climate change emissions”, “health insurance poverty”, and “technology patent” topics for Enacted and Failed bills in both the House and Senate (Fig 4). “Impacts,” “impact,” and “effects” are in House Enacted while “warming,” “global,” and “temperature” are in House Failed, suggesting that, for the House climate change topic, highlighting potential future impacts is associated with enactment while emphasizing increasing global temperatures is associated with failure. In both chambers, “efficiencies” is in Enacted and “variability” is in Failed. In the Senate, “anthropogenic” (human-induced) and “sequestration” (removing greenhouse gases) are in Failed. For the health insurance poverty topic, “medicaid” and “reinsurance” are in both House and Senate Failed. The Senate has words related to more specific health topics, e.g. “immunization” for Failed and “psychiatric” for Enacted. For the patent topic, both chambers have a word related to water (“fish” and “marine”) in the Failed Titles and “geospatial” in the Failed Bodies. Given recent legal developments regarding patenting software, it’s notable that “software” and “computational” are in Failed for the House and Senate, respectively.

**Fig 5:**
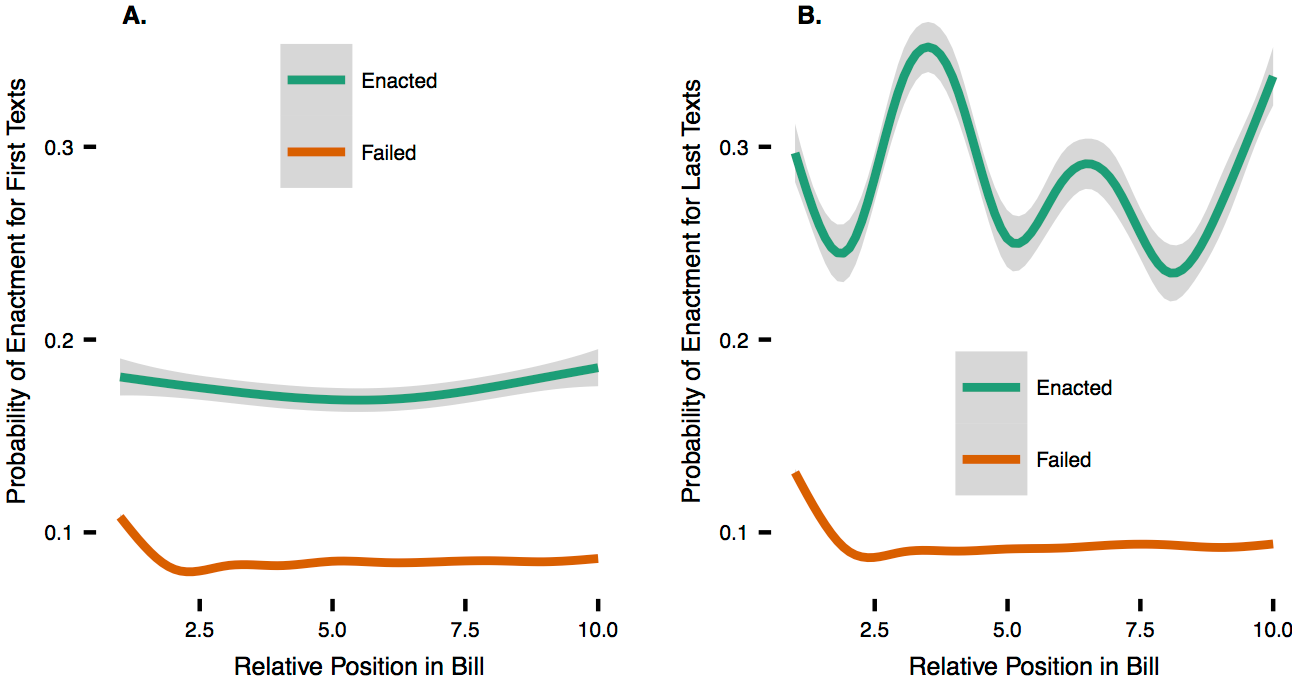
Sentence probabilities across bills for oldest data (A.), newest data (B.). For each bill, we convert the variable length vectors of predicted sentence probabilities to *n*-length vectors by sampling *n* evenly-spaced points from each bill. We set *n = 10* because almost every bill is at least 10 sentences long. Then we loess-smooth the resulting points across all bills to summarize the difference between enacted and failed and newest and oldest texts.

Our language model provides sentence-level predictions for an overall bill and thus predicts what sections of a bill may be the most important for increasing or decreasing the probability of enactment. Fig 5 compares patterns of predicted sentence probabilities as they evolve from the beginning to the end of bills across four categories: enacted and failed and newest and oldest texts. In the newest texts of enacted bills, there is much more variation in predicted probabilities within bills.

**Fig 6:**
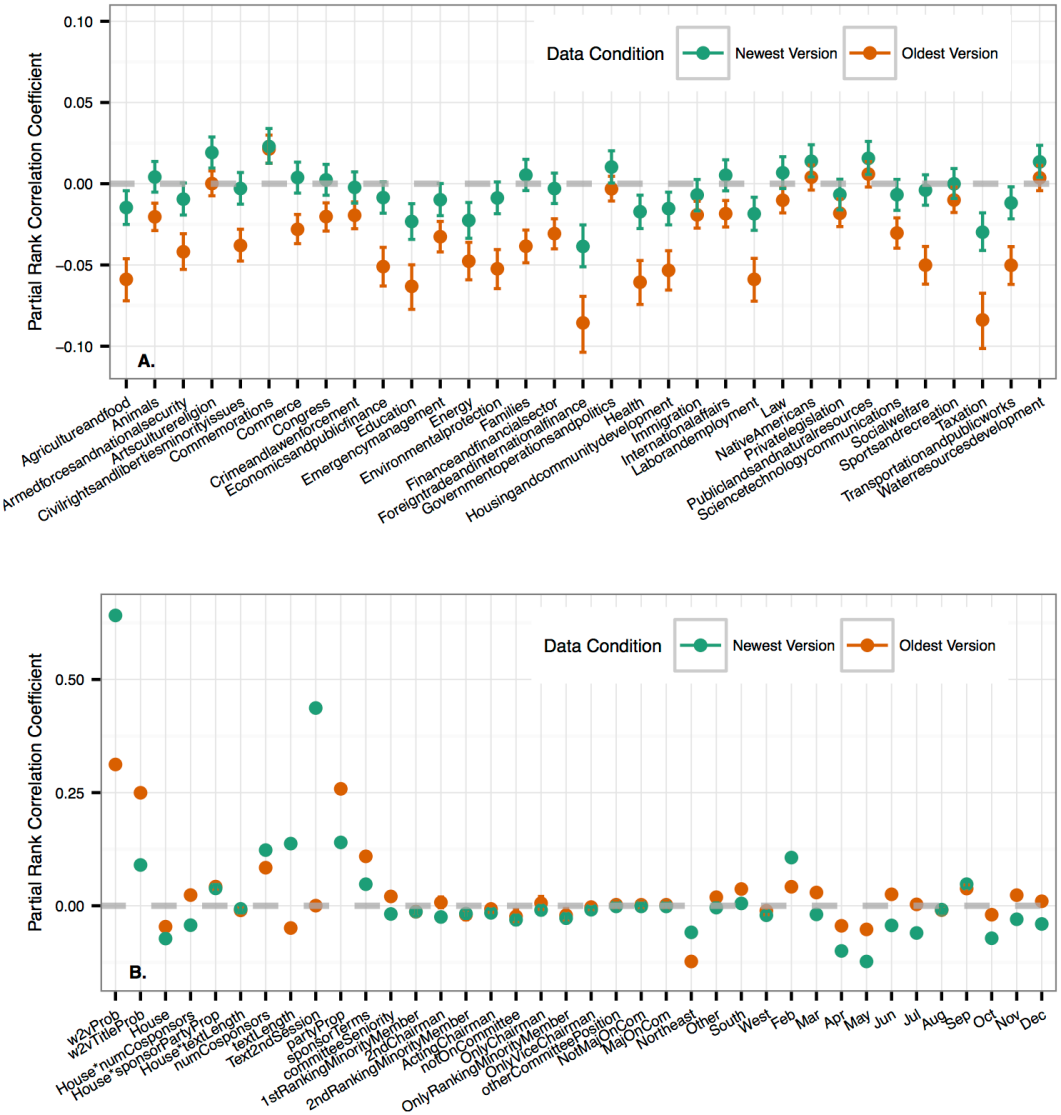
Partial rank correlation coefficient estimates between all *w2vGLM* predictor variables and predicted probabilities (n = 55,695, the subset of the observations used to predict the 113th Congress that had no missing values). Bars represent 95% confidence intervals. **A.** Effects of top subjects. Social Sciences and History is used as the reference subject so no effect is estimated for that factor level. **B.** Effects of all other variables other than subject. January and North Central are the reference levels for the month and region factors. This means they are the base level that is not included as a predictor variable itself—the standard practice when estimating linear models with factor variables. See S1 Appendix for same analysis of *GLM*.

We conducted a partial rank correlation coefficient sensitivity analysis to estimate the effect of each predictor variable on the predicted probability of enactment. These are not bivariate correlations between variables and the predicted probabilities, rather, they are estimates of correlation *after controlling for* the effect of all other predictor variables, e.g. the effect of a bill being introduced in the House is negative after controlling for the other effects in the model (Fig 6B) but bills introduced in the House are enacted at a 0.043 rate while Senate bills are enacted at a 0.025 rate. If we stopped with the simple descriptive statistic we could have incorrectly concluded that introducing a bill in the House will increase its odds, all else equal.

The two subjects with the largest negative effects are Foreign Trade and International Finance, and Taxation (Fig 6A). Some bills fail because their content is integrated into other bills and this is especially true for tax-related bills [2]. With the oldest data model, increasing bill length decreases enactment probability but with the newest data the opposite relationship holds (Fig 6B). We repeated the sensitivity analysis on the model where no text predictions are included (*GLM*, see S1 Appendix), and found that, under both time conditions, when we don’t control for the probability of the text by including our language model predictions (*GLM*), longer texts are more negative than when we control for the text (*w2vGLM*), and that this difference is much larger for the newest data. This suggests that the better we capture the probability of the text and control for its effects, the better we isolate estimates of non-textual effects.

If the bill sponsor’s party is the majority party of their chamber, the probability of the bill is much higher, especially with the oldest data where the model relies on this as a key signal of success. Increasing the number of terms the sponsor has served in Congress also has a positive effect. The predictive model learned interactions as well: the number of co-sponsors has a stronger positive effect in the Senate for the newest data and in the House for the oldest data. If the bill text scored by the language model is in the second session of the Congress, for the newest data model, this can serve as a signal that a bill is being updated and thus it has a higher chance of enactment. For the oldest data, this means the bill was introduced in the second session, which is not particularly indicative of success or failure.

## 4 Discussion

We compared five models across three performance measures and two data conditions over 14 years. A model using only bill text outperforms a model using only bill context for newest data, while context-only outperforms text-only for oldest data. In all conditions text consistently adds predictive power.

In addition to accurate predictions, we are able to improve our understanding of bill content by using a text model designed to explore differences across chamber and enactment status for important topics. Our textual analysis serves as an exploratory tool for investigating subtle distinctions across categories that were previously impossible to investigate at this scale. The same analysis can be applied to any words in the large legislative vocabulary. The global sensitivity analysis of the full model provides insights into the variables affecting predicted probabilities of enactment. For instance, when predicting bills as they are first introduced, the text of the bill and the proportion of the chamber in the bill sponsor’s party have similarly strong positive effects. The full text of the bill is by far the most important predictor when using the most up-to-date data. The oldest data model relies more on title predictions than the newest data model, which is understandable given that titles rarely change after bill introduction. Comparing effects across time conditions and across models not including text suggests that controlling for accurate estimates of the text probability is important for estimating the effects of non-textual variables.

Although the effect estimates are not causal and estimates on predictors correlated with each other may be biased, they represent our best estimates of predictive relationships within a model with the strongest predictive performance and are thus useful for understanding the process of law-making. This methodology can be applied to analyze any predictive model by treating it as a “black-box” data-generating process, therefore predictive power of a model can be optimized and subsequent analysis can uncover interpretable global relationships between predictors and output. Our work provides guidance on effectively combining text and context for prediction *and analysis* of complex systems with highly imbalanced outcomes that are related to textual data. Our system for determining the probability of enactment across the thousands of bills under consideration focuses effort on legislation that is likely to matter, allowing users to identify policy signal amid political and procedural noise.

## Supporting information

S1 Appendix Supplementary information. (PDF)

## References

[1] Martin T, Hofman JM, Sharma A, Anderson A, Watts DJ. Exploring Limits to Prediction in Complex Social Systems. In: Proceedings of the 25th International Conference on World Wide Web. WWW’16. International World Wide Web Conferences Steering Committee; 2016. p. 683–694. Available from: 10.1145/2872427.2883001.

[2] Yano T, Smith NA, Wilkerson JD. Textual Predictors of Bill Survival in Congressional Committees. In: Proceedings of the 2012 Conference of the North American Chapter of the Association for Computational Linguistics: Human Language Technologies. NAACL HLT’12. Association for Computational Linguistics; 2012. p. 793–802. Available from: http://dl.acm.org/citation.cfm?id=2382029.2382157.

[3] Wang E, Liu D, Silva J, Carin L, Dunson DB. Joint Analysis of Time-Evolving Binary Matrices and Associated Documents. In: Lafferty JD, Williams CKI, Shawe-Taylor J, Zemel RS, Culotta A, editors. Advances in Neural Information Processing Systems 23. Curran Associates, Inc.; 2010. p. 2370–2378. Available from: http://papers.nips.cc/paper/4152-joint-analysis-of-time-evolving-binary-matrices-and-associated-documents.pdf.

[4] Gerrish SM, Blei DM. Predicting legislative roll calls from text. In: In Proc. of ICML; 2011.

[5] Wilkerson J, Smith D, Stramp N. Tracing the Flow of Policy Ideas in Legislatures: A Text Reuse Approach. American Journal of Political Science. 2015-10-01;59(4):943–956. 10.1111/ajps.12175

[6] Katz DM, Bommarito MJ, Blackman J. Predicting the Behavior of the Supreme Court of the United States: A General Approach. 2014-07-21;(ID 2463244). 10.1371/journal.pone.0174698 PMC5389610 28403140

[7] Nay JJ. Gov2Vec: Learning Distributed Representations of Institutions and Their Legal Text. In: Proceedings of 2016 EMNLP Workshop on Natural Language Processing and Computational Social Science. Association for Computational Linguistics; 2016. p. 49–54. Available from: http://www.aclweb.org/anthology/W16-5607.

[8] Mikolov T, Sutskever I, Chen K, Corrado GS, Dean J. Distributed Representations of Words and Phrases and their Compositionality. In: Burges CJC, Bottou L, Welling M, Ghahramani Z, Weinberger KQ, editors. Advances in Neural Information Processing Systems 26. Curran Associates, Inc.; 2013. p. 3111–3119. Available from: http://papers.nips.cc/paper/5021-distributed-representations-of-words-and-phrases-and-their-compositionality.pdf.

[9] Bengio Y, Ducharme R, Vincent P, Janvin C. A Neural Probabilistic Language Model. J Mach Learn Res. 2003-3;3:1137–1155.

[10] Rehurek R, Sojka P. Software Framework for Topic Modelling with Large Corpora. In: Proceedings of the LREC 2010 Workshop on New Challenges for NLP Frameworks. ELRA; 2010. p. 45–50.

[11] Taddy M. Document Classification by Inversion of Distributed Language Representations. In: Proceedings of the 53rd Annual Meeting of the Association for Computational Linguistics. vol. Short Papers. Association for Computational Linguistics; 2015-07-31. p. 45–49.

[12] Breiman L, Friedman J, Stone CJ, Olshen RA. Classification and Regression Trees. 1st ed Chapman and Hall/CRC; 1984-1-01.

[13] Friedman JH. Greedy Function Approximation: A Gradient Boosting Machine. The Annals of Statistics. 2001;29(5):1189–1232. 10.1214/aos/1013203451

[14] Breiman L. Random Forests. Machine Learning. 2001-10-01;45(1):5–32. 10.1023/A:1010933404324

[15] www h2o ai. H2O.ai. 2016;.

[16] Breiman L. Stacked Regressions. Machine Learning. 1996-7;24(1):49–64. 10.1023/A:1018046112532

[17] van dL MJ, Polley EC, Hubbard AE. Super Learner. Statistical Applications in Genetics and Molecular Biology. 2007;6(1). 10.2202/1544-6115.1309 17910531

[18] Altman DG, Bland JM. Diagnostic tests 3: receiver operating characteristic plots. BMJ: British Medical Journal. 1994-7-16;309(6948):188 10.1136/bmj.309.6948.188 8044101 PMC2540706

[19] Saltelli A, Annoni P. How to avoid a perfunctory sensitivity analysis. Environmental Modelling & Software. 2010-12;25(12):1508–1517. 10.1016/j.envsoft.2010.04.012

[20] Pujol G, Iooss B, Janon A, Boumhaout K, Da Veiga S, Delage T, et al. sensitivity: Sensitivity Analysis. 2014-08-26. Available at: http://cran.r-project.org/web/packages/sensitivity/index.html

[21] Saltelli A, Chan K, Scott EM. Sensitivity Analysis. 1st ed Wiley; 2009-3-16.

[22] Stewart III C, Woon J. Congressional Committee Assignments, 103rd to 112th Congresses, 1993–2013: Senate [April 11, 2011]; 2011.

[23] Stewart III C, Woon J. Congressional Committee Assignments, 103rd to 113th Congresses, 1993–2015: House [March 9, 2016]; 2016.

[24] Office CB. Letter to the Honorable John Boehner providing an estimate for H.R. 6079, the Repeal of Obamacare Act; 2012. Available from: https://www.cbo.gov/publication/43471.

