# Supplementary material for: Predicting and understanding law-making with word vectors and an ensemble model
Source: PLoS One. 2017 May 10;12(5):e0176999. doi: 10.1371/journal.pone.0176999 (PMC5425031; doi:10.1371/journal.pone.0176999)
Supplement: S1 Appendix — (PDF) [file pone.0176999.s001.pdf]

Supplement for *Predicting and Understanding Law-Making with Machine Learning*

July 6, 2016

Contents

1 Related Work **1**

2 Data **2**

2.1 Data Splitting 2

2.2 Failed ACA-related Bills 2

2.3 Committee Data 16

3 Model Performance **17**

4 Error Analysis **17**

5 Model Analysis **19**

References **22**

1 Related Work

Quantitative political scientists have applied text modeling methods to understand political processes (1–3). The majority of this work is focused on variants of topic modeling (4). There has also been related work on predicting Supreme Court outcomes (5, 6).

Some scholars have addressed prediction of congressional outcomes. (7) predict the roll call votes for the Senate bills in 2008 for which less than 90% of senators agreed. A test of model evaluation has little power when testing on less than one chamber of one year because of potential idiosyncratic Congresses. (8) develop a model that uses the text of bills to predict law-maker's political positions; however this model cannot be used to predict votes on a bill without using other law-maker's votes on the same bill and therefore cannot be used in its current form for forecasting future bill enactment. (9) develop a hybrid topic model-spatial model that can forecast roll-call votes from the text of the bills, using data from 1997 to 2011 (we use 1993 to 2015). It is difficult to compare (9) to our work because (9) predict individual votes of members of Congress rather than whether a bill was enacted. Furthermore, many bills introduced are never brought to a vote.

Most similar to our research is (10) who predict whether a bill will survive consideration by House of Representatives committees, using an l_1 regularized logistic regression model. To incorporate the bill texts, they use binary features indicating the presence of vocabulary terms. They do not use a statistically proper scoring rule, e.g. log loss or brier score, to compare the model to its baseline competitors and they only compare to a baseline model for one chamber of one held-out congress (they report results for their full model on one chamber of three held-out Congresses). The statistically improper performance measure (accuracy) and the small number of testing Congresses reduces the statistical power of their model comparison. With that in mind, they find that using the text alone accounts for the majority of the accuracy of their model.

This is the first study to forecast bill enactment for the House and Senate for multiple Congresses and systematically evaluate forecasting performance. The other work reviewed either models committee recommendation or roll call votes. Only a small proportion of introduced bills are brought to a vote, and whether a bill will be signed into law is the most significant legislative outcome with the largest implications, therefore we directly address this task.

2 Data

2.1 Data Splitting

Starting with the 107th Congress, models are sequentially trained on data from only *previous* Congresses and tested on the *current* Congress. We advance a Congress and repeat until the 113th Congress has served as the test data (Fig. S 1). We complete this process with the language models and save the predictions. Then, for the full model, we repeat the process. Starting with the 107th Congress, models are sequentially trained on data from only *previous* Congresses and tested on the "current" Congress, where the data includes the predictions made by the language models for all the bills in a Congress. This simulates real-time deployment of the model. Only data available before the bill's final outcome is known is used in the model to make a prediction, and only data from previous Congresses is used for training models.

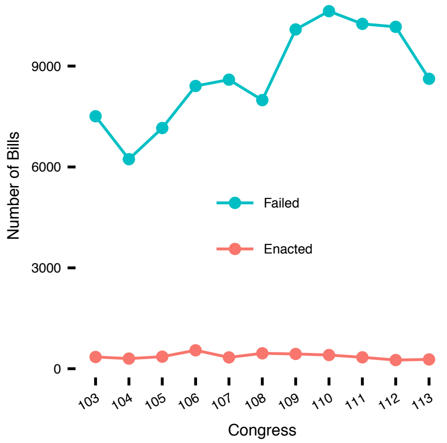

Fig. S1. Number of bills enacted and failed for Congresses 103 (1993-1995) through 113 (2013-2015).

2.2 Failed ACA-related Bills

Below we list the 296 bills with "Patient Protection and Affordable Care Act" in the title that were not enacted. These bills were predicted by our model to have very low probability of being enacted.

- To amend the Patient Protection and Affordable Care Act to ensure appropriate treatment of Department of Veterans Affairs and Department of Defense health programs.
- To repeal the Patient Protection and Affordable Care Act.
- To repeal the Patient Protection and Affordable Care Act.
- To repeal the Patient Protection and Affordable Care Act and enact the Empowering Patients First Act in order to provide incentives to encourage health insurance coverage.
- To repeal specific provisions in the Patient Protection and Affordable Care Act.
- To repeal the Patient Protection and Affordable Care Act.
- To repeal the Patient Protection and Affordable Care Act and to replace such Act with incentives to encourage health insurance coverage, and for other purposes.
- To amend the Patient Protection and Affordable Care Act to provide for participation in the Exchange of the President, Vice-President, Members of Congress, political appointees, and congressional staff.

- To eliminate sweetheart deals under the Patient Protection and Affordable Care Act.
- To repeal the Patient Protection and Affordable Care Act.
- To amend the Patient Protection and Affordable Care Act to clarify the coverage for congressional employees through Exchanges under title I of such Act.
- To repeal the provisions of the Patient Protection and Affordable Care Act providing for the Independent Payment Advisory Board.
- To repeal the Patient Protection and Affordable Care Act.
- To repeal the Patient Protection and Affordable Care Act and the Health Care and Education Reconciliation Act of 2010 and enact the OPTION Act of 2009.
- To amend the Patient Protection and Affordable Care Act to modify special rules relating to coverage of abortion services under such Act.
- To repeal provisions of the Patient Protection and Affordable Care Act relating to health savings accounts, and for other purposes.
- To amend title 5, United States Code, to extend eligibility for coverage under the Federal Employees Health Benefits Program with respect to certain adult dependents of Federal employees and annuitants, in conformance with amendments made by the Patient Protection and Affordable Care Act.
- To repeal the Patient Protection and Affordable Care Act.
- To amend the Patient Protection and Affordable Care Act to clarify that the Act does not affect standards or procedures in medical malpractice actions.
- To amend section 1877 of the Social Security Act to delay by 2 years the expansion cut-off date imposed by the Patient Protection and Affordable Care Act.
- To provide for extension of COBRA continuation coverage until coverage is available otherwise under either an employment-based health plan or through an American Health Benefit Exchange under the Patient Protection and Affordable Care Act.
- To amend titles XIX and XVIII of the Social Security Act, as amended by the Patient Protection and Affordable Care Act and the Health Care and Education Reconciliation Act of 2010, with respect to payment of disproportionate share hospitals (DSH) under the Medicare and Medicaid programs.
- To repeal the Patient Protection and Affordable Care Act and the Health Care and Education Reconciliation Act of 2010, repeal the 7.5 percent threshold on the deduction for medical expenses, provide for increased funding for high-risk pools, allow acquiring health insurance across State lines, and allow for the creation of association health plans.
- To repeal the Patient Protection and Affordable Care Act and the Health Care and Education Reconciliation Act of 2010 and enact the Common Sense Health Care Reform and Affordability Act.
- To amend the Patient Protection and Affordable Care Act to permit a State to elect not to establish an American Health Benefit Exchange.
- To repeal certain provisions of the Patient Protection and Affordable Care Act relating to the limitation on the Medicare exception to the prohibition on certain physician referrals for hospitals and to transparency reports and reporting of physician ownership or investment interests.
- To amend the Internal Revenue Code of 1986 to repeal the Patient Protection and Affordable Care Act and the Health Care and Education Reconciliation Act of 2010 and to replace it with provisions reforming the health care system by putting patients back in charge of health care.
- To provide that no funds are authorized to be appropriated to the Internal Revenue Service to expand its workforce in order to implement, enforce, or otherwise carry out either the Patient Protection and Affordable Care Act or the Health Care and Education Reconciliation Act of 2010.
- To amend title I of the Patient Protection and Affordable Care Act to permit certain individuals losing their COBRA continuation coverage to have access to the high-risk health insurance pool program established under such title.
- To amend the Patient Protection and Affordable Care Act to expedite the application of the provision prohibiting rescissions of health insurance coverage.
- To repeal a provision of the Patient Protection and Affordable Care Act providing for funds to a health care facility and rescind funds made available under such section.
- To amend the Patient Protection and Affordable Care Act to establish a public health insurance option.
- To deauthorize appropriation of funds to carry out the Patient Protection and Affordable Care Act and the Health Care and Education Reconciliation Act of 2010.

- To amend the Patient Protection and Affordable Care Act to repeal certain limitations on tax health care benefits.
- To repeal limitations imposed by the Patient Protection and Affordable Care Act on health-related tax benefits under the Internal Revenue Code of 1986 and to treat high deductible health plans as qualified health plans under such Act.
- To preserve Medicare beneficiary choice by repealing section 3204(a) of the Patient Protection and Affordable Care Act.
- To amend title I of the Patient Protection and Affordable Care Act to provide for appropriate procedures under such title for verification of citizenship status.
- To preserve Medicare beneficiary choice by restoring and expanding the Medicare open enrollment and disenrollment opportunities repealed by section 3204(a) of the Patient Protection and Affordable Care Act.
- To repeal the Patient Protection and Affordable Care Act and the Health Care and Education Reconciliation Act of 2010.
- To amend the Patient Protection and Affordable Care Act to protect rights of conscience with regard to requirements for coverage of specific items and services.
- A bill to repeal the Patient Protection and Affordable Care Act.
- A bill to repeal the Patient Protection and Affordable Care Act.
- A bill to amend the Patient Protection and Affordable Care Act to provide for participation in the Exchange of the President, Vice-President, Members of Congress, political appointees, and congressional staff.
- A bill to repeal unwarranted provisions from the Patient Protection and Affordable Care Act and to more efficiently use taxpayer dollars in health care spending.
- A bill to amend title 5, United States Code, to extend eligibility for coverage under the Federal Employees Health Benefits Program with respect to certain adult dependents of Federal employees and annuitants, in conformance with amendments made by the Patient Protection and Affordable Care Act.
- A bill to provide for extension of COBRA continuation coverage until coverage is available otherwise under either an employment-based health plan or through an American Health Benefit Exchange under the Patient Protection and Affordable Care Act.
- A bill to amend the Patient Protection and Affordable Care Act to repeal certain limitations on tax health care benefits.
- A bill to promote transparency and accountability concerning the implementation of the Patient Protection and Affordable Care Act.
- A bill to allow an earlier start for State health care coverage innovation waivers under the Patient Protection and Affordable Care Act.
- A bill to preserve Medicare beneficiary choice by restoring and expanding the Medicare open enrollment and disenrollment opportunities repealed by section 3204(a) of the Patient Protection and Affordable Care Act.
- A bill to provide for an earlier start for State health care coverage innovation waivers under the Patient Protection and Affordable Care Act, and for other purposes.
- To repeal the Patient Protection and Affordable Care Act and related health-care provisions and to enact in its place incentives to encourage health insurance coverage, and for other purposes.
- To amend the Patient Protection and Affordable Care Act to permit a State to elect not to establish an American Health Benefit Exchange.
- To deauthorize appropriation of funds to carry out the Patient Protection and Affordable Care Act and the Health Care and Education Reconciliation Act of 2010.
- To repeal the Patient Protection and Affordable Care Act and the Health Care and Education Reconciliation Act of 2010.
- To repeal the Patient Protection and Affordable Care Act (Public Law 111-148) and related health-care provisions.
- To amend the Patient Protection and Affordable Care Act to establish a public health insurance option.
- To repeal the Patient Protection and Affordable Care Act and title I of the Health Care and Education Reconciliation Act of 2010 while preserving the reauthorization of the Indian Health Care Improvement Act.

- To repeal the Patient Protection and Affordable Care Act and the Health Care and Education Reconciliation Act of 2010, repeal the 7.5 percent threshold on the deduction for medical expenses, provide for increased funding for high-risk pools, allow acquiring health insurance across State lines, and allow for the creation of association health plans.
- To amend titles XIX and XVIII of the Social Security Act, as amended by the Patient Protection and Affordable Care Act and the Health Care and Education Reconciliation Act of 2010, with respect to payment of disproportionate share hospitals (DSH) under the Medicare and Medicaid programs.
- To amend the Patient Protection and Affordable Care Act to provide for participation in the Exchange of the President, Vice-President, Members of Congress, political appointees, and congressional staff.
- To repeal the Patient Protection and Affordable Care Act and to take meaningful steps to lower health care costs and increase access to health insurance coverage without raising taxes, cutting Medicare benefits for seniors, adding to the national deficit, intervening in the doctor-patient relationship, or instituting a government takeover of health care.
- To repeal title I of the Patient Protection and Affordable Care Act and to amend the Public Health Service Act to provide for cooperative governing of individual health insurance coverage offered in interstate commerce.
- To repeal the Patient Protection and Affordable Care Act and the Health Care and Education Reconciliation Act of 2010 and to take meaningful steps to lower health care costs and increase access to health insurance coverage without raising taxes, cutting Medicare benefits for seniors, adding to the national deficit, intervening in the doctor-patient relationship, or instituting a government takeover of health care.
- To repeal the Patient Protection and Affordable Care Act and the health care-related provisions in the Health Care and Education Reconciliation Act of 2010 and to amend title 5, United States Code, to establish a national health program administered by the Office of Personnel Management to offer Federal employee health benefits plans to individuals who are not Federal employees, and for other purposes.
- To provide for an earlier start for State health care coverage innovation waivers under the Patient Protection and Affordable Care Act, and for other purposes.
- To repeal limitations imposed by the Patient Protection and Affordable Care Act on health-related tax benefits under the Internal Revenue Code of 1986 and to treat high deductible health plans as qualified health plans under such Act.
- To amend the Internal Revenue Code of 1986 to repeal the provisions of the Patient Protection and Affordable Care Act that limit distributions from medical-related tax-preferred accounts for medicines only if the medicines are prescribed drugs or insulin and to repeal the increase in additional tax on distributions from health savings accounts and Archer MSAs not used for qualified medical expenses.
- To repeal certain provisions in the Patient Protection and Affordable Care Act related to patient centered outcomes research and rescind unobligated appropriations related to such provisions and to repeal certain health care-related provisions in the American Recovery and Reinvestment Act of 2009 and rescind unobligated appropriations related to such provisions for purposes of reducing the national debt.
- To repeal the information reporting requirements added by the Patient Protection and Affordable Care Act.
- To amend the Patient Protection and Affordable Care Act to repeal certain limitations on health care benefits.
- To deauthorize and rescind funding for the Patient Protection and Affordable Care Act and health-care-related provisions of the Health Care and Education Reconciliation Act of 2010.
- To enable States to opt out of certain provisions of the Patient Protection and Affordable Care Act.
- To prevent the Patient Protection and Affordable Care Act from establishing health care provider standards of care in medical malpractice or medical product liability cases, and for other purposes.
- To allow an earlier start for State health care coverage innovation waivers under the Patient Protection and Affordable Care Act.
- To amend title I of the Patient Protection and Affordable Care Act to provide for a process for waiver of requirements of that title where the requirement is asserted to otherwise result in a significant decrease in access to coverage or significant increase in premiums or other costs.

- To provide for expedited consideration by the Supreme Court of certain actions challenging the constitutionality of certain provisions of the Patient Protection and Affordable Care Act.
- To repeal certain provisions of the Patient Protection and Affordable Care Act relating to the limitation on the Medicare exception to the prohibition on certain physician referrals for hospitals and to transparency reports and reporting of physician ownership or investment interests.
- To amend the Patient Protection and Affordable Care Act to protect rights of conscience with regard to requirements for coverage of specific items and services.
- To provide for fiscal accountability for new direct funding under the Patient Protection and Affordable Care Act by converting its direct funding into authorizations of appropriations and by rescinding unobligated direct funding.
- To eliminate sweetheart deals under the Patient Protection and Affordable Care Act.
- To repeal the annual fee on health insurance providers enacted by the Patient Protection and Affordable Care Act.
- To preserve Medicare beneficiary choice by restoring and expanding the Medicare open enrollment and disenrollment opportunities repealed by section 3204(a) of the Patient Protection and Affordable Care Act.
- To repeal mandatory funding provided to States in the Patient Protection and Affordable Care Act to establish American Health Benefit Exchanges.
- To amend title I of the Patient Protection and Affordable Care Act to provide for appropriate procedures under such title for verification of citizenship status.
- To amend subtitle D of title I of the Patient Protection and Affordable Care Act to clarify Congressional consent to and expand flexibility for interstate health choice compacts.
- To amend the Patient Protection and Affordable Care Act to provide for greater disclosure in the process for waiving annual limitation requirements under that Act.
- To amend the Patient Protection and Affordable Care Act to repeal distributions for medicine qualified only if for prescribed drug or insulin.
- To firewall the Medicare Trusts Funds by restoring to those Trust Funds funds transferred by the Patient Protection and Affordable Care Act.
- To amend the Patient Protection and Affordable Care Act to have Early Innovator grant funds returned by States apply towards deficit reduction.
- To amend the Patient Protection and Affordable Care Act to provide State flexibility for the offering of health benefits through alternative health arrangements.
- To amend title I of the Patient Protection and Affordable Care Act to expand access to high risk pools.
- To amend section 1343 of the Patient Protection and Affordable Care Act to ensure the privacy of individually identifiable health information in connection with risk adjustment.
- To amend the Patient Protection and Affordable Care Act to modify special rules relating to coverage of abortion services under such Act.
- To amend titles XIX and XXI of the Social Security Act, titles I and II of the Patient Protection and Affordable Care Act, and other Acts for the purpose of eliminating certain health entitlement programs and reducing the deficit.
- To amend title XXVII of the Public Health Service Act to apply to retiree-only health plans the extension of dependent health coverage for individuals through 26 years of age provided for by the Patient Protection and Affordable Care Act.
- To enable States to opt out of the Medicaid expansion-related provisions of the Patient Protection and Affordable Care Act.
- To amend the Internal Revenue Code of 1986 to provide that the prohibition on suits to restrain assessment or collection of tax does not apply to the tax provisions of the Patient Protection and Affordable Care Act or the Health Care and Education Reconciliation Act of 2010.
- To amend title XVIII of the Social Security Act to repeal the reduction in Medicare disproportionate share hospital (DSH) payments made by section 3133 of the Patient Protection and Affordable Care Act.
- To repeal the Patient Protection and Affordable Care Act and provide for comprehensive health reform, and for other purposes.
- To repeal the provisions of the Patient Protection and Affordable Care Act providing for the Independent

Payment Advisory Board.

- To repeal the Patient Protection and Affordable Care Act and the Health Care and Education Reconciliation Act of 2010, to amend the Internal Revenue Code of 1986 to repeal the percentage floor on medical expense deductions, expand the use of tax-preferred health care accounts, and establish a charity care credit, to amend the Social Security Act to create a Medicare Premium Assistance Program and reform EMTALA requirements, and to amend the Public Health Service Act to provide for cooperative governing of individual and group health insurance coverage offered in interstate commerce.
- To repeal the Patient Protection and Affordable Care Act, to amend the Public Health Service Act to provide individual and group market reforms to protect health insurance consumers, and for other purposes.
- To amend title I of the Patient Protection and Affordable Care Act to ensure that the coverage offered under multi-State qualified health plans offered in Exchanges is consistent with the Federal abortion funding ban.
- To amend the Internal Revenue Code of 1986 to repeal the amendments made by the Patient Protection and Affordable Care Act which disqualify expenses for over-the-counter drugs under health savings accounts and health flexible spending arrangements.
- To repeal section 4004 of the Patient Protection and Affordable Care Act (authorizing an education and outreach campaign).
- To repeal the provisions of the Patient Protection and Affordable Care Act and the health-related provisions of the Health Care and Education Reconciliation Act of 2010 not declared unconstitutional by the Supreme Court.
- To prohibit funding to implement any provision of the Patient Protection and Affordable Care Act or of the health-related provisions of the Health Care and Education Reconciliation Act of 2010.
- To repeal the Patient Protection and Affordable Care Act and health care-related provisions in the Health Care and Education Reconciliation Act of 2010.
- To repeal provisions of the Patient Protection and Affordable Care Act relating to health savings accounts, and for other purposes.
- To amend the Patient Protection and Affordable Care Act to provide for savings to the Federal Government by permitting pass-through funding for State authorized public entity health benefits pools.
- To amend the Patient Protection and Affordable Care Act so as to eliminate the authority of the Secretary of Health and Human Services to limit the ability of medical providers to conduct lawful business, and for other purposes.
- To amend the Patient Protection and Affordable Care Act to improve eligibility requirements for uninsured individuals with a preexisting condition for coverage under the Preexisting Condition Insurance Program (PCIP).
- To provide that the individual mandate under the Patient Protection and Affordable Care Act shall not be construed as a tax.
- To amend subtitle B of title I of the Patient Protection and Affordable Care Act to extend the temporary high-risk insurance pool program to the territories.
- A bill to repeal the Patient Protection and Affordable Care Act.
- A bill to provide for an earlier start for State health care coverage innovation waivers under the Patient Protection and Affordable Care Act, and for other purposes.
- A bill to repeal the sunset of the Economic Growth and Tax Relief Reconciliation Act of 2001 with respect to the expansion of the adoption credit and adoption assistance programs, to repeal the sunset of the Patient Protection and Affordable Care Act with respect to increased dollar limitations for such credit and programs, and to allow the adoption credit to be claimed in the year expenses are incurred, regardless of when the adoption becomes final.
- A bill to amend the Patient Protection and Affordable Care Act to provide for participation in the Exchange of the President, Vice President, Members of Congress, political appointees, and congressional staff.
- A bill to enable States to opt out of certain provisions of the Patient Protection and Affordable Care Act.
- A bill to allow an earlier start for State health care coverage innovation waivers under the Patient

Protection and Affordable Care Act.

- A bill to amend the Patient Protection and Affordable Care Act to repeal certain limitations on health care benefits.
- A bill to amend the Patient Protection and Affordable Care Act to repeal distributions for medicine qualified only if for prescribed drug or insulin.
- A bill to ensure that all Americans have access to waivers from the Patient Protection and Affordable Care Act.
- A bill to amend the Patient Protection and Affordable Care Act to protect rights of conscience with regard to requirements for coverage of specific items and services.
- A bill to amend the Patient Protection and Affordable Care Act to authorize additional funding for the pregnancy assistance fund.
- A bill to enable States to opt out of the Medicaid expansion-related provisions of the Patient Protection and Affordable Care Act.
- A bill to amend the Internal Revenue Code of 1986 and the Patient Protection and Affordable Care Act to extend, expand, and improve the qualifying therapeutic discovery project program.
- A bill to provide that the individual mandate under the Patient Protection and Affordable Care Act shall not be construed as a tax.
- To repeal the Patient Protection and Affordable Care Act and the Health Care and Education Reconciliation Act of 2010.
- To repeal portions of the Patient Protection and Affordable Care Act, to reduce Federal Government spending and to reduce the salaries of Members of Congress, and for other purposes.
- To amend subtitle B of title I of the Patient Protection and Affordable Care Act to extend the temporary high-risk insurance pool program to the territories.
- To amend the Patient Protection and Affordable Care Act to establish a public health insurance option.
- To amend title I of the Patient Protection and Affordable Care Act to ensure that the coverage offered under multi-State qualified health plans offered in Exchanges is consistent with the Federal abortion funding ban.
- To repeal the provisions of the Patient Protection and Affordable Care Act providing for the Independent Payment Advisory Board.
- To delay until 2016 provisions of the Patient Protection and Affordable Care Act scheduled to take effect in 2014 or 2015 and to delay the application of sequestration until 2014.
- To repeal title I of the Patient Protection and Affordable Care Act and to amend the Public Health Service Act to provide for cooperative governing of individual health insurance coverage offered in interstate commerce.
- To repeal the annual fee on health insurance providers enacted by the Patient Protection and Affordable Care Act.
- To repeal the Patient Protection and Affordable Care Act and the health care-related provisions in the Health Care and Education Reconciliation Act of 2010 and to amend title 5, United States Code, to establish a national health program administered by the Office of Personnel Management to offer Federal employee health benefits plans to individuals who are not Federal employees, and for other purposes.
- To amend the Patient Protection and Affordable Care Act to protect rights of conscience with regard to requirements for coverage of specific items and services, to amend the Public Health Service Act to prohibit certain abortion-related discrimination in governmental activities, and for other purposes.
- To deauthorize appropriation of funds, and to rescind unobligated appropriations, to carry out the Patient Protection and Affordable Care Act and the Health Care and Education Reconciliation Act of 2010.
- To amend the Patient Protection and Affordable Care Act to provide for savings to the Federal Government by permitting pass-through funding for State authorized public entity health benefits pools.
- To amend the Patient Protection and Affordable Care Act to repeal certain limitations on health care benefits.
- To repeal provisions of the Patient Protection and Affordable Care Act relating to health savings accounts, and for other purposes.

- To amend section 1101 of the Patient Protection and Affordable Care Act to provide additional funds to permit additional individuals to enroll under the preexisting condition insurance program and expand eligibility, to be funded through a temporary increase in the cigarette tax, and for other purposes.
- To amend the Patient Protection and Affordable Care Act to provide for participation in the Exchange of the President, Vice President, and Executive cabinet officials in same manner as Members of Congress and Congressional staff.
- To provide that the only health plans that the Federal Government may make available to the President, Vice President, Members of Congress, and Federal employees are those created under the Patient Protection and Affordable Care Act or offered through a health insurance exchange.
- To provide for an earlier start for State health care coverage innovation waivers under the Patient Protection and Affordable Care Act, and for other purposes.
- To repeal certain provisions of the Patient Protection and Affordable Care Act relating to the premium tax credits and cost-sharing subsidies.
- To amend the Patient Protection and Affordable Care Act to provide for participation in the Exchange of the President, Vice President, Members of Congress, political appointees, and Congressional staff.
- To prohibit the Secretary of the Treasury from enforcing the Patient Protection and Affordable Care Act and the Health Care and Education Reconciliation Act of 2010.
- To amend the Patient Protection and Affordable Care Act to apply to Delegates and Resident Commissioners to the Congress, and to employees of committees and leadership offices of Congress, the requirement of such Act that the only health plans that the Federal Government may make available to Members of Congress and congressional staff are plans created or offered through an Exchange established under such Act.
- To prohibit the implementation or enforcement of any requirement of the Patient Protection and Affordable Care Act until certifications are made that taxpayer information is not and will not be used for targeting any individual or group that provides information to the Internal Revenue Service for political reasons or on the basis of political views, and for other purposes.
- To prohibit Federal funds for the establishment or operation of patient navigator programs under the Patient Protection and Affordable Care Act, and for other purposes.
- To repeal the Patient Protection and Affordable Care Act and health care-related provisions in the Health Care and Education Reconciliation Act of 2010.
- To amend the Public Health Service Act to provide individual and group market reforms to protect health insurance consumers, to make such reforms and protections contingent on the enactment of legislation repealing the Patient Protection and Affordable Care Act, and for other purposes.
- To preserve Medicare beneficiary choice by restoring and expanding the Medicare open enrollment and disenrollment opportunities repealed by section 3204(a) of the Patient Protection and Affordable Care Act.
- To amend the Internal Revenue Code of 1986 to modify the definition of applicable large employer for purposes of the employer mandate in the Patient Protection and Affordable Care Act.
- To prohibit the funding of the Patient Protection and Affordable Care Act.
- To amend title I of the Patient Protection and Affordable Care Act to provide for a process for waiver of requirements of that title where the requirement is asserted to otherwise result in a significant decrease in access to coverage or significant increase in premiums or other costs.
- To delay enrollment in qualified health plans in State or Federally facilitated Exchanges until 1 year after final rules are published establishing the verification and other procedures to be used to implement section 1411 of the Patient Protection and Affordable Care Act and carrying out sections 6055 and 6056 of the Internal Revenue Code of 1986.
- To delay the application of the Patient Protection and Affordable Care Act.
- To amend the Patient Protection and Affordable Care Act so as to eliminate the authority of the Secretary of Health and Human Services to limit the ability of medical providers to conduct lawful business, and for other purposes.
- To amend the Internal Revenue Code of 1986 to repeal the amendments made by the Patient Protection and Affordable Care Act which disqualify expenses for over-the-counter drugs under health savings accounts and health flexible spending arrangements.
- To prohibit for a one-year period beginning September 30, 2013, the implementation, operation, and

coordination of a Federal Data Services Hub or any similar database system for determining or verifying eligibility under the Patient Protection and Affordable Care Act.

- To amend the Internal Revenue Code of 1986 to provide veterans with a 1-year exemption from the requirement to maintain minimum essential coverage under the Patient Protection and Affordable Care Act.
- To discontinue eligibility of former Members of Congress and their dependents for coverage under the Federal Employees Health Benefit Program (FEHBP) if the Patient Protection and Affordable Care Act is repealed.
- To repeal the Patient Protection and Affordable Care Act and the Health Care and Education Reconciliation Act of 2010; to amend the Internal Revenue Code of 1986 to repeal the percentage floor on medical expense deductions, expand the use of tax-preferred health care accounts, and establish a charity care credit; to amend the Social Security Act to create a Medicare Premium Assistance Program, reform EMTALA requirements, and to replace the Medicaid program and the Children's Health Insurance program with a block grant to the States; to amend the Public Health Service Act to provide for cooperative governing of individual and group health insurance coverage offered in interstate commerce; and for other purposes.
- To prevent the implementation of certain tax and fee provisions of the Patient Protection and Affordable Care Act until the Secretary of the Treasury certifies that reporting requirements relating to employer status and employee income levels and health care status may be made with 100 percent accuracy and without fraud.
- To provide that certain requirements of the Patient Protection and Affordable Care Act do not apply if the American Health Benefit Exchanges are not operating on October 1, 2013.
- To delay for one year certain amendments to the Medicaid program made by the Patient Protection and Affordable Care Act, and for other purposes.
- To require certain certifications before funds may be awarded for the operation of a Navigator program under the Patient Protection and Affordable Care Act, and for other purposes.
- To amend the Internal Revenue Code of 1986 to modify the definition of full-time employee for purposes of the employer mandate in the Patient Protection and Affordable Care Act.
- To amend the Patient Protection and Affordable Care Act to eliminate the limitation on deductibles for employer-sponsored health plans.
- To amend the Patient Protection and Affordable Care Act to prohibit a government subsidy or contribution for the premiums of a health plan by a Member of Congress or Members' staff or congressional leadership or committee staff.
- To amend the Patient Protection and Affordable Care Act to prohibit a government subsidy for the purchase of a health plan by a Member of Congress.
- To amend the Patient Protection and Affordable Care Act to provide that no Government contribution may be made toward the cost of Exchange coverage for any Member of Congress or congressional staff.
- To amend the Patient Protection and Affordable Care Act with respect to health insurance coverage for certain congressional staff and political appointees in the executive branch, and for other purposes.
- To exclude individuals who receive health insurance coverage pursuant to the terms of a collective bargaining agreement from tax credits and reductions in cost-sharing under the Patient Protection and Affordable Care Act.
- To clarify the application of all laws, including the Patient Protection and Affordable Care Act, to the Federal Government and Congress, and for other purposes.
- To prohibit the Secretary of the Treasury from enforcing the Patient Protection and Affordable Care Act and the Health Care and Education Reconciliation Act of 2010.
- To repeal the Patient Protection and Affordable Care Act and related reconciliation provisions, to promote patient-centered health care, and for other purposes.
- To amend the Patient Protection and Affordable Care Act to prohibit a government subsidy for the purchase of a health plan by a Member of Congress.
- To clarify the application of all laws, including the Patient Protection and Affordable Care Act, to the Federal Government and Congress, and for other purposes.
- To repeal the Patient Protection and Affordable Care Act and to take meaningful steps to lower health care costs and increase access to health insurance coverage without raising taxes, cutting Medicare

benefits for seniors, adding to the national deficit, intervening in the doctor-patient relationship, or instituting a government takeover of health care.

- To provide that the only health plans that the Federal Government may make available to Federal employees responsible for the administration of the Patient Protection and Affordable Care Act are those created under such Act or offered through a health insurance exchange, and for other purposes.
- To amend the Patient Protection and Affordable Care Act to prohibit government subsidies for the purchase of health plans by Members of Congress and congressional staff and to apply to Delegates and Resident Commissioners to the Congress, and to employees of committees and leadership offices of Congress, the requirement of such Act that the only health plans that the Federal Government may make available to Members of Congress and congressional staff are plans created or offered through an Exchange established under such Act.
- To amend the Patient Protection and Affordable Care Act to provide, because of problems relating to the operation of Exchanges, for a hardship exemption from the individual mandate for months of noncoverage, and for other purposes.
- To amend the Patient Protection and Affordable Care Act to provide for health insurance coverage for the President through an Exchange in the same manner as for Members of Congress.
- To provide for an exemption from the individual mandate under the Patient Protection and Affordable Care Act for individuals residing in States in which the Exchange Websites are not fully functional, and for other purposes.
- To provide for a delay of the individual mandate under the Patient Protection and Affordable Care Act until the American Health Benefit Exchanges are functioning properly.
- To amend section 9010 of the Patient Protection and Affordable Care Act to delay the application of the health insurance provider annual fee until 2016 and to provide a process to return to consumers any amounts attributable to the expected application of the annual fee to 2014 or 2015.
- To amend the Patient Protection and Affordable Care Act to ensure that individuals can keep their health insurance coverage.
- To require any communication using Federal funds to advertise or educate the public on certain provisions of the Patient Protection and Affordable Care Act and the Healthcare and Education Reconciliation Act of 2010 to state that such communication was produced at taxpayer expense, and for other purposes.
- To amend the Patient Protection and Affordable Care Act to delay the individual health insurance mandate and any penalties for violating the individual mandate until after there is a certification that the healthcare.gov website is fully operational, and for other purposes.
- To protect personal and financial information by requiring certain certifications by entities awarded funds under the Patient Protection and Affordable Care Act for the operation of a Navigator program or certain other Exchange activities.
- To amend the Patient Protection and Affordable Care Act to allow individuals to opt out of the minimum required health benefits by permitting health insurance issuers to offer qualified health plans that offer alternative benefits to the minimum essential health benefits otherwise required, and for other purposes.
- To amend section 1341 of the Patient Protection and Affordable Care Act to repeal the funding mechanism for the transitional reinsurance program in the individual market, and for other purposes.
- To amend the Patient Protection and Affordable Care Act to delay the individual health insurance mandate and any penalties for violating the individual mandate until after there is a certification that the healthcare.gov or other applicable State Exchange website is fully operational, and for other purposes.
- To clarify the application of all laws, including the Patient Protection and Affordable Care Act, to the Federal Government and Congress, and for other purposes.
- To amend the Patient Protection and Affordable Care Act to permit insurers to offer catastrophic coverage plans to anyone, and for other purposes.
- To enable States to opt out of certain provisions of the Patient Protection and Affordable Care Act.
- To repeal the Patient Protection and Affordable Care Act and provide for comprehensive health reform, and for other purposes.
- To amend title 18, United States Code, to provide for penalties for aggravated identity theft facilitated by employment at an agency implementing the Patient Protection and Affordable Care Act.

- To amend the Patient Protection and Affordable Care Act to prohibition on payment of bonuses and pay increases for executives of a State Exchange funded through Federal grants, and for other purposes.
- To ensure that emergency services volunteers are not counted as full-time employees under the shared responsibility requirements contained in the Patient Protection and Affordable Care Act.
- To require an Exchange established under the Patient Protection and Affordable Care Act to notify individuals in the case that personal information of such individuals is known to have been acquired or accessed as a result of a breach of the security of any system maintained by the Exchange.
- To provide that the reinsurance fee for the transitional reinsurance program under the Patient Protection and Affordable Care Act be applied equally to all health insurance issuers and group health plans.
- To amend section 1101 of the Patient Protection and Affordable Care Act to extend for one year the high risk health insurance pool program, and for other purposes.
- To require notifications by the Secretary of Health and Human Services to Congress and to individuals of breaches of personally identifiable information of such individuals maintained, submitted to, or submitted by a system maintained by Exchanges under the Patient Protection and Affordable Care Act, and for other purposes.
- To repeal sections 1341 and 1342 of the Patient Protection and Affordable Care Act.
- To require the Secretary of Homeland Security the responsibility to develop and provide to the Secretary of Health and Human Services risk-based, performance-based cybersecurity standards for the Federal information technology requirements under the Patient Protection and Affordable Care Act, including the healthcare.gov website, and for other purposes.
- To provide for the repeal of the Patient Protection and Affordable Care Act if it is determined that the Act has resulted in increasing the number of uninsured individuals.
- To repeal sections 1341 and 1342 of the Patient Protection and Affordable Care Act, and for other purposes.
- To amend the Patient Protection and Affordable Care Act to prohibit Government contributions under the Federal employees health benefit program towards Exchange health insurance coverage of Members of Congress.
- To require notification of individuals of breaches of personally identifiable information through Exchanges under the Patient Protection and Affordable Care Act.
- To amend section 1311(a) of the Patient Protection and Affordable Care Act to provide for the recoupment and reallocation of unspent State grant funds, and for other purposes.
- To amend the Patient Protection and Affordable Care Act to require transparency in the operation of American Health Benefit Exchanges.
- To amend title I of the Patient Protection and Affordable Care Act concerning the notice requirements regarding the extent of health plan coverage of abortion.
- To sunset funding under sections 1341 and 1342, and to repeal section 1343, of the Patient Protection and Affordable Care Act, and for other purposes.
- To amend the Patient Protection and Affordable Care Act to eliminate Exchange cost-sharing subsidies, to amend title XVIII of the Social Security Act to create a Medicare Advantage Improvement Fund, and for other purposes.
- To amend title XVIII of the Social Security Act to require Medicare Advantage organizations to disclose certain information on the changes made to the MA plan offered by such organization pursuant to changes required by the Patient Protection and Affordable Care Act and the Health Care and Education Reconciliation Act of 2010, and for other purposes.
- To amend the Patient Protection and Affordable Care Act to authorize the extension of the initial open enrollment period for up to 1 month, and for other purposes.
- To amend the Internal Revenue Code of 1986 to allow employers to exempt employees with health coverage under TRICARE or the Veterans Administration from being taken into account for purposes of the employer mandate under the Patient Protection and Affordable Care Act.
- To amend title I of the Patient Protection and Affordable Care Act to impose restrictions on the risk corridor program.
- To amend the Internal Revenue Code of 1986 to repeal the 30-hour threshold for classification as a full-time employee for purposes of the employer mandate in the Patient Protection and Affordable Care Act and replace it with 40 hours.

- To clarify the treatment under the Patient Protection and Affordable Care Act of health plans in which expatriates are the primary enrollees, and for other purposes.
- To amend the Patient Protection and Affordable Care Act to require States with failed American Health Benefit Exchanges to reimburse the Federal Government for amounts provided under grants for the establishment and operation of such Exchanges.
- To direct the Office of the Actuary of the Centers for Medicare & Medicaid Services and the Comptroller General of the United States to study the impact of the Patient Protection and Affordable Care Act on small businesses.
- To amend the Patient Protection and Affordable Care Act to provide for a temporary shift in the scheduled collection of the transitional reinsurance program payments.
- To direct the Secretary of Health and Human Services to conduct outreach efforts to provide certain health insurance information to individuals enrolled in qualified health plans offered through an Exchange established under title I of the Patient Protection and Affordable Care Act or State plans under the Medicaid program under title XIX of the Social Security Act, and for other purposes.
- To amend title XVIII of the Social Security Act to repeal rebasing of payments for home health services, as required under the Patient Protection and Affordable Care Act, and to replace such rebasing with a Medicare home health value-based purchasing program, and for other purposes.
- To amend title XIX of the Social Security Act to end the increased Federal funding for Medicaid expansion with respect to inmates' hospital care under the Patient Protection and Affordable Care Act, to apply the savings towards a 2015 Medicare Advantage stabilization program to help protect seniors' choices, and for other purposes.
- To amend the Patient Protection and Affordable Care Act to repeal the risk corridor program, and for other purposes.
- To amend the Patient Protection and Affordable Care Act to eliminate benefits under the Federal Employees Health Benefits Program for Members of Congress so they are treated the same way as other taxpayers, and for other purposes.
- To amend the Patient Protection and Affordable Care Act to allow sole proprietors and the spouses and domestic partners of sole proprietors to purchase insurance on the small business exchange, and for other purposes.
- To amend the Public Health Service Act to limit rescissions of coverage under health plans in the individual and group market, contingent on the enactment of legislation repealing the Patient Protection and Affordable Care Act, and for other purposes.
- To amend the Public Health Service Act to prohibit application of preexisting condition exclusions and to guarantee availability of health insurance coverage in the individual and group market, contingent on the enactment of legislation repealing the Patient Protection and Affordable Care Act, and for other purposes.
- To amend the Public Health Service Act to extend health plan coverage to dependent children in the individual and group market, contingent on the enactment of legislation repealing the Patient Protection and Affordable Care Act, and for other purposes.
- To amend the Internal Revenue Code of 1986 to provide for the determination of the employer mandate under the Patient Protection and Affordable Care Act without regard to alien agricultural seasonal workers.
- To amend the Patient Protection and Affordable Care Act to provide privacy protections that enable certain individuals to remove their profiles from the healthcare.gov website, and for other purposes.
- A bill to amend the Patient Protection and Affordable Care Act to authorize additional funding for the pregnancy assistance fund.
- A bill to amend title I of the Patient Protection and Affordable Care Act to ensure that the coverage offered under multi-State qualified health plans offered in Exchanges is consistent with the Federal abortion funding ban.
- A bill to repeal the Patient Protection and Affordable Care Act and the Health Care and Education Reconciliation Act of 2010 entirely.
- A bill to repeal the provisions of the Patient Protection and Affordable Care Act of providing for the Independent Payment Advisory Board.
- A bill to repeal the Patient Protection and Affordable Care Act.

- A bill to provide that the individual mandate under the Patient Protection and Affordable Care Act shall not be construed as a tax.
- A bill to repeal the annual fee on health insurance providers enacted by the Patient Protection and Affordable Care Act.
- A bill to amend the Patient Protection and Affordable Care Act to repeal certain limitations on health care benefits.
- A bill to amend the Internal Revenue Code of 1986 to modify the definition of full-time employee for purposes of the individual mandate in the Patient Protection and Affordable Care Act.
- A bill to provide for an earlier start for State health care coverage innovation waivers under the Patient Protection and Affordable Care Act, and for other purposes.
- A bill to amend the Patient Protection and Affordable Care Act to apply the provisions of the Act to certain Congressional staff and members of the executive branch.
- A bill to prohibit amounts made available by the Patient Protection and Affordable Care Act and the Health Care and Education Reconciliation Act of 2010 from being transferred to the Internal Revenue Service for implementation of such Acts.
- A bill to prohibit the Secretary of the Treasury from enforcing the Patient Protection and Affordable Care Act and the Health Care and Education Reconciliation Act of 2010.
- A bill to provide that certain requirements of the Patient Protection and Affordable Care Act do not apply if the American Health Benefit Exchanges are not operating on October 1, 2013.
- A bill to amend the Patient Protection and Affordable Care Act to clarify provisions with respect to church plans.
- A bill to amend the Internal Revenue Code of 1986 to modify the definition of full-time employee for purposes of the individual mandate in the Patient Protection and Affordable Care Act.
- A bill to amend the Patient Protection and Affordable Care Act to protect rights of conscience with regard to requirements for coverage of specific items and services, to amend the Public Health Service Act to prohibit certain abortion-related discrimination in governmental activities, and for other purposes.
- A bill to provide that certain requirements of the Patient Protection and Affordable Care Act do not apply if the American Health Benefit Exchanges are not operating on October 1, 2013.
- A bill to prohibit the funding of the Patient Protection and Affordable Care Act.
- A bill to prohibit the Secretary of the Treasury from enforcing the Patient Protection and Affordable Care Act and the Health Care and Education Reconciliation Act of 2010.
- A bill to repeal the provisions of the Patient Protection and Affordable Care Act providing for the Independent Payment Advisory Board.
- A bill to delay the implementation of the employer responsibility provisions of the Patient Protection and Affordable Care Act.
- A bill to condition the provision of premium and cost-sharing subsidies under the Patient Protection and Affordable Care Act upon a certification that a program to verify household income is operational.
- A bill to limit the availability of tax credits and reductions in cost-sharing under the Patient Protection and Affordable Care Act to individuals who receive health insurance coverage pursuant to the provisions of a Taft-Hartley plan.
- A bill to delay the application of the Patient Protection and Affordable Care Act.
- A bill to amend the Patient Protection and Affordable Care Act to apply the provisions of the Act to certain Congressional staff and members of the executive branch.
- A bill to ensure that the personal and private information of Americans enrolling in Exchanges established under the Patient Protection and Affordable Care Act is secured with proper privacy and data security safeguards.
- A bill to correct inconsistencies in the definitions relating to Native Americans in the Patient Protection and Affordable Care Act.
- A bill to amend the Patient Protection and Affordable Care Act to require transparency in the operation of American Health Benefit Exchanges.
- A bill to provide for a delay of the individual mandate under the Patient Protection and Affordable Care Act until the American Health Benefit Exchanges are functioning properly.
- A bill to amend the Patient Protection and Affordable Care Act to ensure that individuals can keep their health insurance coverage.

- A bill to amend the Patient Protection and Affordable Care Act to repeal distributions for medicine qualified only if for prescribed drug or insulin.
- A bill to amend the Patient Protection and Affordable Care Act to improve the patient navigator program.
- A bill to delay the implementation of the individual health coverage mandate under the Patient Protection and Affordable Care Act.
- A bill to amend the Patient Protection and Affordable Care Act to extend the initial open enrollment period.
- A bill to enable States to opt out of certain provisions of the Patient Protection and Affordable Care Act.
- A bill to clarify that the anti-kickback laws apply to qualified health plans, the federally-facilitated marketplaces, and other plans and programs under title I of the Patient Protection and Affordable Care Act, and for other purposes.
- A bill to provide that the reinsurance fee for the transitional reinsurance program under the Patient Protection and Affordable Care Act be applied equally to all health insurance issuers and group health plans.
- A bill to amend the Patient Protection and Affordable Care Act to provide further options with respect to levels of coverage under qualified health plans.
- A bill to ensure that emergency services volunteers are not counted as full-time employees under the shared responsibility requirements contained in the Patient Protection and Affordable Care Act.
- A bill to amend the Patient Protection and Affordable Care Act to provide for a fixed annual open enrollment period.
- A bill to provide for the repeal of the Patient Protection and Affordable Care Act if it is determined that the Act has resulted in increasing the number of uninsured individuals.
- A bill to require notification of individuals of breaches of personally identifiable information through Exchanges under the Patient Protection and Affordable Care Act.
- A bill to provide for the repeal of certain provisions of the Patient Protection and Affordable Care Act that have the effect of rationing health care.
- A bill to amend the Internal Revenue Code of 1986 to modify provisions relating to determinations of full-time equivalent employees for purposes of the Patient Protection and Affordable Care Act.
- A bill to amend the Patient Protection and Affordable Care Act to provide a permanent path for the direct enrollment of individuals in qualified health plans.
- A bill to amend the Patient Protection and Affordable Care Act to provide greater flexibility in offering health insurance coverage across State lines.
- A bill to amend the Patient Protection and Affordable Care Act to enhance access for independent agents and brokers to information regarding marketplace enrollment.
- A bill to revise reporting requirements under the Patient Protection and Affordable Care Act to preserve the privacy of individuals, and for other purposes.
- A bill to amend the Internal Revenue Code of 1986 to allow employers to exempt employees with health coverage under TRICARE or the Veterans Administration from being taken into account for purposes of the employer mandate under the Patient Protection and Affordable Care Act.
- A bill to amend the Patient Protection and Affordable Care Act to provide for a temporary shift in the scheduled collection of the transitional reinsurance program payments.
- A bill to amend the Patient Protection and Affordable Care Act so as to eliminate the authority of the Secretary of Health and Human Services to limit the ability of medical providers to conduct lawful business, and for other purposes.
- A bill to amend the Patient Protection and Affordable Care Act to require States with failed American Health Benefit Exchanges to reimburse the Federal Government for amounts provided under grants for the establishment and operation of such Exchanges.
- A bill to direct the Office of the Actuary of the Centers for Medicare & Medicaid Services and the Comptroller General of the United States to study the impact of the Patient Protection and Affordable Care Act on small businesses.
- A bill to require the Congressional Budget Office to annually report changes in direct spending and revenue associated with the Patient Protection and Affordable Care Act.

2.3 Committee Data

For our *committeePosition* variable, we needed to report one *committeePosition* for every bill to model the effect of the bill sponsor's committee position on enactment, but for some bills there may be more than one committee that a bill is assigned to and the bill sponsor is on. If this occurs, we use the lowest number on the "leadership codes" list below to select one leadership position of the sponsor on a committee the bill is assigned to. Committee membership data was downloaded from web.mit.edu/17.251/www/data_page.html (11, 12).

Code	Factor
0	Inapplicable
11	Only Chairman
12	1st Chairman
13	2nd Chairman
14	3rd Chairman
16	Acting Chairman
21	Only ranking minority member
22	1st ranking minority member
23	2nd ranking minority member
24	3rd ranking minority member
31	Only Speaker
32	1st Speaker
33	2nd Speaker
41	Only Majority Leader
42	1st Majority Leader
43	2nd Majority Leader
44	3rd Majority Leader
51	Only Majority Whip
52	1st Majority Whip
53	2nd Majority Whip
61	Only Minority Leader
62	1st Minority Leader
63	2nd Minority Leader
64	Only Minority Whip
65	1st Minority Whip
66	2nd Minority Whip
81	Only Vice Chairman
82	1st Vice Chairman
83	2nd Vice Chairman
86	Co-chairman

According to (12), the committee seniority variable is computed as follows: "For the House, this is the number of the term presently served by the member on the committee. Each time a member leaves the committee and returns, the number of terms is reset to 1. For the Senate, the unit of measure is the year. It can best be described as the year on the committee as of the assignment date for that entry. For example, a freshman senator assigned at the beginning of a congress to committee would have this variable coded 1. Similarly, a senator assigned to a committee in October of the 2nd session would have that initial entry coded 1. If he is reappointed to the same committee at the start of the next congress, he is still coded 1 since he is still serving his first year on the committee."

3 Model Performance

Fig. S 2 visualizes how the two most frequently applied binary classification probability scoring functions, the log score and the brier score, assign performance scores based on predicted probabilities and actual outcomes. If a model assigns high probability to an unsuccessful bill it is penalized more than if it was less confident and, conversely, if a model assigns high probability to a successful bill it is rewarded more than if it was not confident (13).

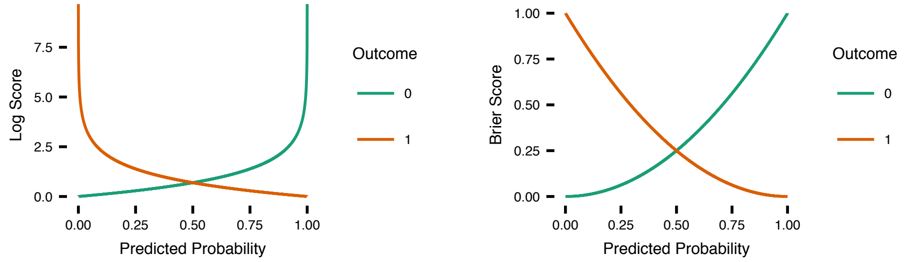

Fig. S2. Log and brier scores as a function of predicted probabilities and actual outcomes. A lower score is better.

The majority of the density of the predicted probabilities for all bills is peaked around 0.01 and the predicted probabilities for the ACA and ARRA were > 0.5 (Fig. S 3).

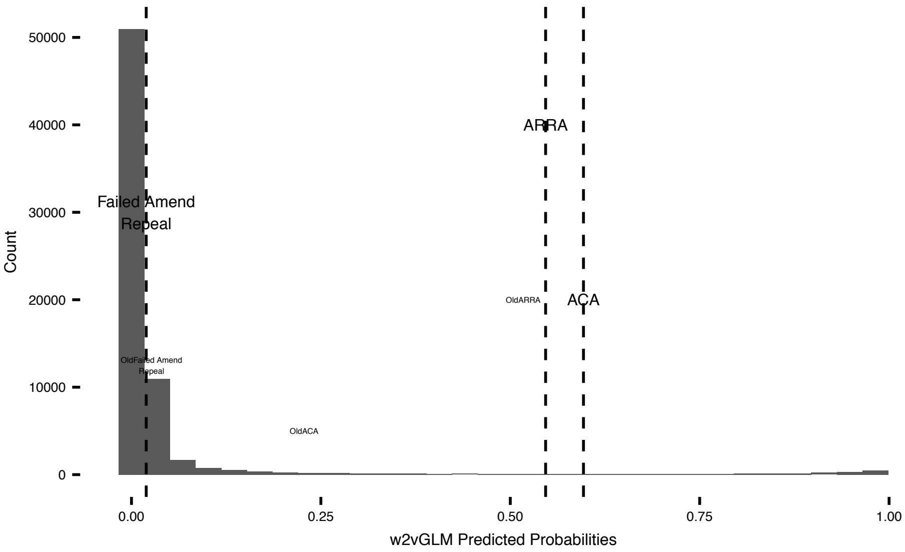

Fig. S3. Predicted probabilities for all *w2vGLM* predictions with newest bills and labeled predicted probabilities of two controversial enacted bills and the mean of the predicted probabilities for the bills attempting to repeal or amend the ACA that were not enacted, which is in the peak of the probability density with most other failed bills. The dashed lines and large font labels correspond to predictions with the newest versions and the small font labels correspond to predictions with the oldest versions.

4 Error Analysis

We fit linear regression models to the relationship between the subjects of bills and the log loss of our predictions of the bills to learn where the model performs the worst. The larger the estimated coefficient, the worse the model (*w2v* in Fig. S 4 and *w2vGLM* in Fig. S 5) performs for that subject category.

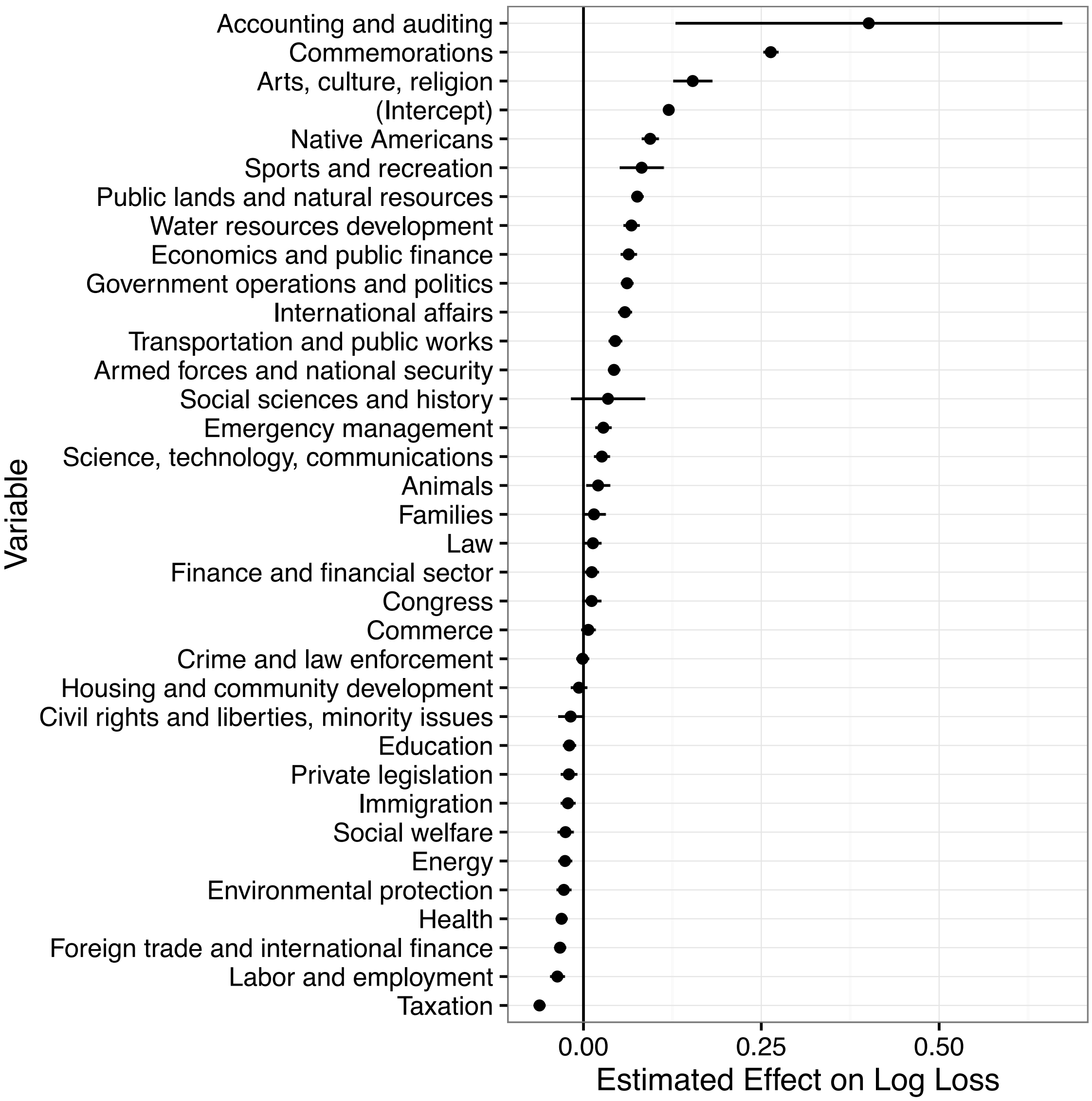

Fig. S4. Ordinary least squares regression model predicting log loss in *w2v* predictions. Positive coefficients suggest that the text model performs worse when the bill is on that subject.

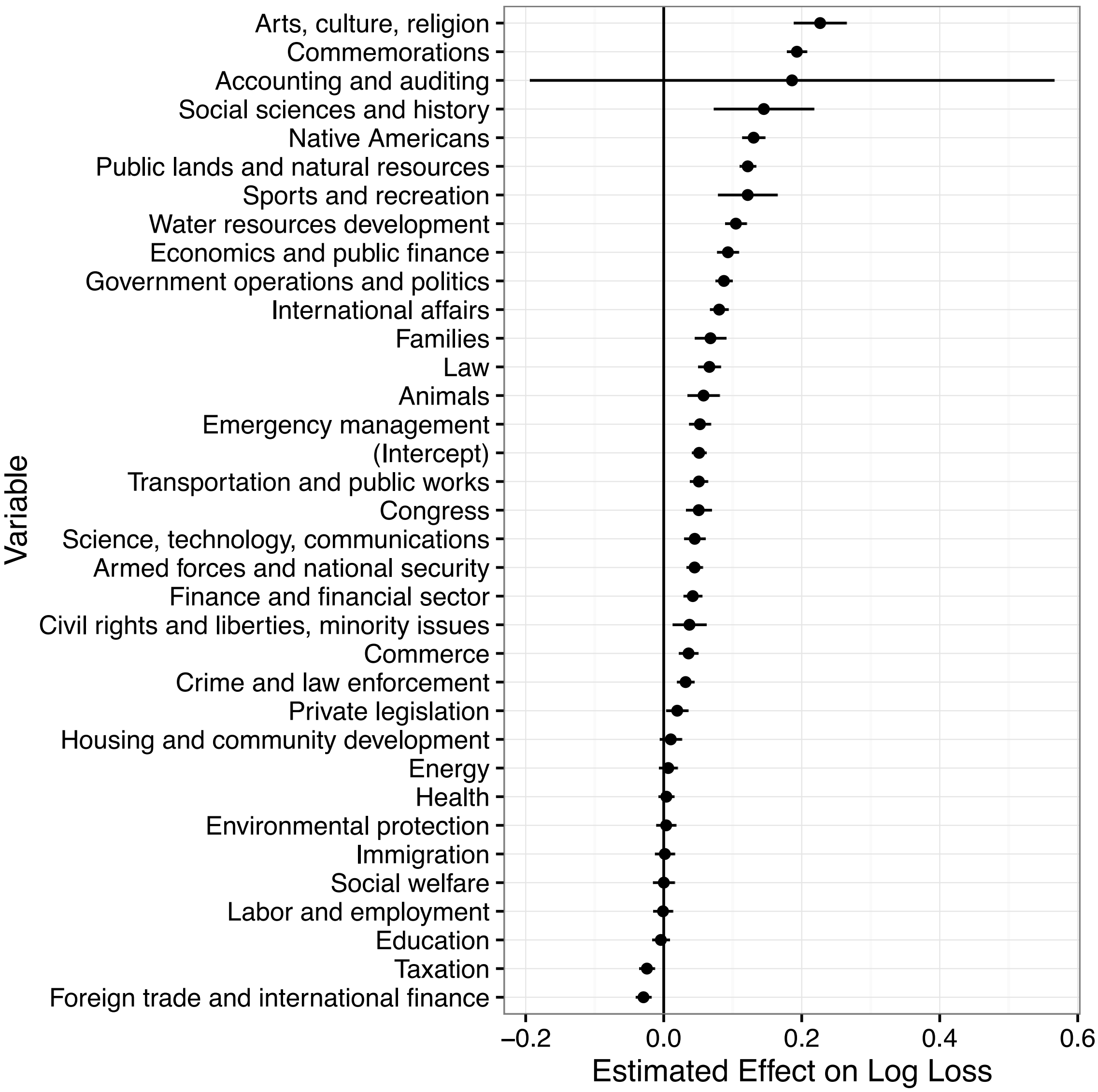

Fig. S5. Ordinary least squares regression model predicting log loss in *w2vGLM* predictions. Positive coefficients suggest that the full model performs worse when the bill is on that subject.

5 Model Analysis

We compare predicted probabilities between pure textual (*w2v*) and context (*GLM*) models. The largest positive difference in mean predicted probabilities across subject categories (with more than 100 observations) between *w2v* and *GLM* is the "Armed forces and national security" subject, where the *w2v* mean predicted probabilities are 0.08 *higher* than the *GLM* mean predicted probabilities for those bills. This suggests that the contextual factors associated with most bills classified by Congress as primarily Armed Forces and National Security related are making them significantly less likely to be enacted than their full text would warrant.

Fig. S9. Loess-smoothed bivariate relationships between predictors and *w2vGLM* predicted probabilities with oldest data.

References

1. Grimmer J (2010) A bayesian hierarchical topic model for political texts: Measuring expressed agendas in senate press releases. *Political Analysis* 18(1):1–35.

2. Grimmer J, Stewart BM (2013) Text as data: The promise and pitfalls of automatic content analysis methods for political texts. *Political Analysis* 21(3):267–297.

3. Roberts ME, et al. (2014) Structural topic models for open-ended survey responses. *American Journal of Political Science* 58(4):1064–1082.

4. Blei DM, Ng AY, Jordan MI (2003) Latent dirichlet allocation. *J Mach Learn Res* 3:993–1022.

5. Ruger TW, Kim PT, Martin AD, Quinn KM (2004) The supreme court forecasting project: Legal and political science approaches to predicting supreme court decisionmaking. *Columbia Law Review* 104(4):1150–1210.

6. Katz DM, Bommarito MJ, Blackman J (2014) *Predicting the behavior of the supreme court of the united states: A general approach* (Social Science Research Network, Rochester, NY) Available at: http://papers.ssrn.com/abstract=2463244 [Accessed June 22, 2016].

7. Wang E, Liu D, Silva J, Carin L, Dunson DB (2010) Joint analysis of time-evolving binary matrices and associated documents. *Advances in Neural Information Processing Systems 23*, eds Lafferty JD, Williams CKI, Shawe-Taylor J, Zemel RS, Culotta A (Curran Associates, Inc.), pp 2370–2378.

8. Gerrish S, Blei DM (2012) How they vote: Issue-adjusted models of legislative behavior. *Advances in Neural Information Processing Systems 25*, eds Pereira F, Burges CJC, Bottou L, Weinberger KQ (Curran Associates, Inc.), pp 2753–2761.

9. Gerrish SM, Blei DM (2011) Predicting legislative roll calls from text. *In Proc. of ICML.*

10. Yano T, Smith NA, Wilkerson JD (2012) Textual predictors of bill survival in congressional committees. *Proceedings of the 2012 Conference of the North American Chapter of the Association for Computational Linguistics: Human Language Technologies*, NAACL HLT '12. (Association for Computational Linguistics, Stroudsburg, PA, USA), pp 793–802.

11. Stewart III C, Woon J (2011) Congressional committee assignments, 103rd to 112th congresses, 1993-2013: Senate [april 11, 2011].

12. Stewart III C, Woon J (2016) Congressional committee assignments, 103rd to 113th congresses, 1993-2015: House [march 9, 2016].

13. Good IJ (1952) Rational decisions. *Journal of the Royal Statistical Society Series B (Methodological)* 14(1):107–114.
